# Decoding the Gut–Fat–Heart Axis: From Molecular Communication Networks to Clinical Translation Strategies

**DOI:** 10.3390/ijms27125596

**Published:** 2026-06-20

**Authors:** Zijin Sun, Wei Shao, Haojia Zhang, Kai Wang, Yongchao Liu, Rui Zhou

**Affiliations:** 1 School of Traditional Chinese Medicine, Beijing University of Chinese Medicine, Beijing 102488, China; 2 School of Chinese Medicine, Beijing University of Chinese Medicine, Beijing 102488, China

**Keywords:** holistic metabolic perspective, gut–fat–heart axis, trimethylamine N-oxide, lipotoxicity, clinical translation strategies, cardiovascular disease

## Abstract

The prevention and treatment of cardiovascular disease (CVD) are undergoing a paradigm shift from a lipid-centric approach to a holistic metabolic perspective. Central to this evolution is the gut–fat–heart axis, a sophisticated three-dimensional communication network that integrates neural, endocrine, and immunometabolic signaling to regulate systemic lipid homeostasis. This manuscript systematically explores how the gut microbiota acts as a “metabolic organ” to remotely control host health through the production of bioactive metabolites and the modulation of molecular communication networks. At the physiological level, microbial products such as short-chain fatty acids (SCFAs) and modified bile acids regulate energy balance and lipid synthesis via the FXR-FGF15/19 axis and G protein-coupled receptors. Furthermore, gut hormones like GLP-1 and neuro-reflex pathways involving the vagus nerve provide rapid control over postprandial lipid clearance and feeding behavior. Conversely, pathological dysbiosis triggers the accumulation of harmful metabolites, such as trimethylamine N-oxide (TMAO) and lipopolysaccharides (LPS), which drive lipotoxicity, vascular inflammation, and “dysfunctional HDL” formation. These processes accelerate the progression of atherosclerosis, heart failure, and metabolic syndrome. Finally, the article outlines promising clinical translation strategies, including the development of TMA lyase inhibitors, next-generation probiotics, and the use of phytochemicals to reshape the microbial landscape. By decoding the molecular dialogues within the gut–fat–heart axis, this research provides a novel strategic vantage point for the integrated management of cardiovascular–kidney–metabolic (CKM) syndrome.

## 1. Introduction

### 1.1. From the “Cholesterol Hypothesis” to the “Holistic Metabolic Perspective”

For a long time, the prevention and treatment of cardiovascular disease (CVD) have primarily focused on controlling low-density lipoprotein cholesterol (LDL-C) and triglycerides. However, as research has deepened, the academic community has gradually recognized that simply regulating lipid levels often fails to fully eliminate residual cardiovascular risk. The modern medical paradigm is shifting toward a “holistic metabolic perspective.” This shift is particularly evident in the recently proposed concept of the “Cardiovascular–Kidney–Metabolic (CKM) Syndrome,” which emphasizes the shared pathophysiological basis linking obesity, diabetes, chronic kidney disease (CKD), and cardiovascular disease. This commonality is characterized by dysfunctional adipose tissue and systemic lipid metabolism disorders [1]. Within this complex pathological state, the accumulation of intracellular lipid intermediates and glucose is perceived by the body as energy excess. This triggers impaired autophagy, mitochondrial dysfunction, and increased oxidative stress, collectively accelerating the progression of cardiomyopathy and metabolic diseases [2]. Therefore, focusing solely on blood lipid concentrations is insufficient to explain the full picture of the disease. Attention must be directed toward the ectopic deposition of lipids in target organs (such as the heart and kidneys) and the resulting “lipotoxicity” [3].

Against this backdrop, the concept of the “holobiont” emerged, suggesting that human metabolic health results from the combined actions of the host genome and symbiotic microbial genomes. As the hub of this metabolic network, the gut not only handles the initial processing of dietary carbohydrates and lipids, but its barrier function and metabolic products also directly influence lipid homeostasis in the liver and throughout the body [4]. Research indicates that gut microbiota dysbiosis is closely associated with systemic lipid metabolism disorders, and interventions targeting the gut microbiome have demonstrated potential for multi-organ protection. For instance, enriching beneficial bacterial communities (such as Alistipes and Anaerotruncus) can significantly improve lipid metabolism, suppress hepatic de novo lipogenesis, and enhance fatty acid oxidation, thereby alleviating systemic insulin resistance and inflammation [5].

Moreover, molecular communication networks play a pivotal role in maintaining this cross-organ metabolic homeostasis. Neurotransmitters such as gamma-aminobutyric acid (GABA) exert effects not only within the central nervous system but also through signaling in the pancreas and gut. By enhancing gut microbiota diversity and alleviating intestinal inflammation, GABA signaling improves systemic lipid profiles, thereby exerting protective effects on cardiac and renal systems [6]. Simultaneously, the discovery of novel biomarkers like copeptin further reveals potential mechanisms of neurohormonal signaling in regulating glucose and lipid metabolism, linking metabolic syndrome to cardiovascular risk [7]. Targeting the regulation of lipid droplet dynamics and intervening in the gut microbiota–metabolite axis are emerging as critical approaches to overcome limitations in traditional lipid-lowering therapies and establish novel strategies for CVD prevention and treatment [2,3].

### 1.2. The Gut: The Hidden Mastermind of Cardiovascular Lipid Homeostasis

**Figure 1:**
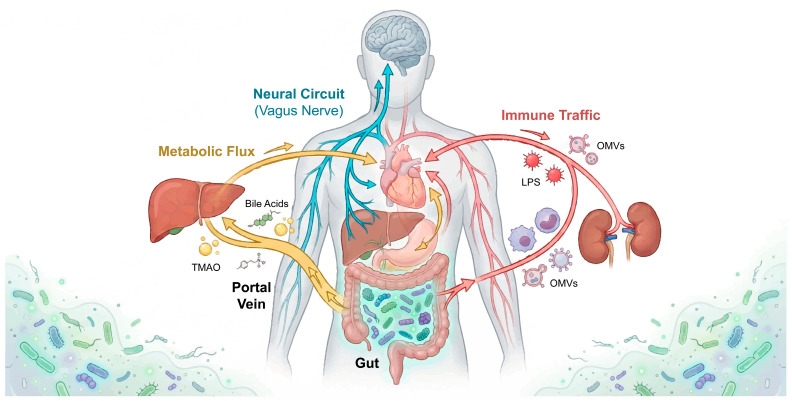
The “Gut–Lipid–Heart” Superorganism Communication Network: A Holobiont View. The human body outline illustrates the three primary pathways (coded by color) by which the gut microbiota remotely controls the brain, heart, and kidneys. **Neural Circuit [blue arrows]**: Represents neuronal signals originating from the gut (lower-middle). Arrows travel along the vagus nerve, with branches directing signals to both the brain (top) and the heart (upper-middle), highlighting microbial influence on neurological and cardiovascular regulation and systemic energy balance. **Metabolic Flux [golden arrows]**: Represents the transport of gut-derived metabolites. Compounds flow from the gut via the portal vein to the liver. From the liver, arrows branch to deliver metabolites to the heart and the brain. **Immune Traffic [red arrows]**: Represents the dissemination of immune-mediating factors. Originating from the gut, arrows carry bacterial-derived pro-inflammatory components systemically to the heart and pass through the kidney region. Specific amplified elements include outer membrane vesicles (OMVs), lipopolysaccharide (LPS), and various types of leukocyte immune cells (amplified). **Solid color-coded arrows (blue, golden, and red)** indicate the predominant direction of signaling (neuronal signals, metabolic flux, and immune traffic, respectively) between the gut microbiota and the target organs. TMAO, trimethylamine N-oxide; LPS, lipopolysaccharide; OMVs, outer membrane vesicles.

From a modern medical perspective, the “gut–fat–heart axis” transcends traditional nutrient absorption pathways and is now defined as a complex three-dimensional network involving metabolic signaling, immune activation, and cross-organ communication. Based on the Remote Sensing and Signaling Theory (RSST), the gut maintains homeostasis of endogenous small-molecule metabolites through close collaboration with organs such as the liver and kidneys [8,9] (Figure 1). Within this network, solute carrier (SLC) and ATP-binding cassette (ABC) transporter superfamilies serve as pivotal nodes. They not only facilitate the transport of drugs and toxins but also regulate the transmembrane flow of bile acids, fatty acids, and gut microbiota metabolites under physiological conditions, thereby optimizing the body’s overall metabolic efficiency [10,11]. Notably, organic anion transporters (OATs) within the SLC22 family have been identified as pivotal hubs linking the gut microbiota to the host metabolic system. By processing diverse signaling molecules, including short-chain fatty acids, they mediate “inter-organismal” communication mechanisms [11].

The gut microbiota, acting as the “invisible manipulator” of this axis, directly intervenes in the host’s lipid and energy metabolism by generating specific metabolites. Research indicates that the aryl hydrocarbon receptor (AHR) plays a “master regulator” role in this process [12]. AHR not only regulates the expression of specific transporters and metabolic enzymes but also broadly influences biochemical pathways involving fatty acids, bile acids, and microbial products (such as indole derivatives), determining the distribution and clearance of these hydrophobic metabolites across tissues [12]. This cross-scale metabolic remodeling capability demonstrates that gut-derived signaling molecules can transcend anatomical boundaries to remotely regulate the physiological environment of the cardiovascular system. When renal function is impaired, this finely tuned homeostasis network undergoes reset. Gut-derived uremic toxins, such as indole-3-carbinol sulfate and p-cresol sulfate, fail to be effectively cleared and instead accumulate as pathological signaling molecules within the body [13,14].

At the pathophysiological level, the association between gut microbiota dysbiosis and cardiovascular disease is mediated precisely through these accumulated metabolic toxins. Intestinal protein-bound toxins, which should be excreted via renal OAT1 and OAT3 transporters, become retained in the bloodstream when renal function declines. By inducing oxidative stress and inflammatory responses, they directly contribute to pathological processes such as cardiac remodeling, left ventricular hypertrophy, and fibrosis [15]. This “gut–kidney–heart” pathological crosstalk reveals that the gut is not only a site of lipid absorption but also a source of key signaling molecules regulating systemic vascular health. Altered tryptophan metabolism pathways and abnormal fluctuations in their derivatives (e.g., kynurenine, kynurenic acid) further confirm that gut metabolites play a decisive role in maintaining or disrupting cardiovascular lipid homeostasis through OATs-mediated “long-range sensing” networks [13].

### 1.3. The Gut Microbiota as a Central Hub for Multi-Organ Metabolic Homeostasis

The gut microbiome is no longer viewed merely as an auxiliary to digestion, but rather as a central hub regulating systemic metabolic homeostasis and inter-organ communication. In the epidemiology of metabolic dysfunction-associated fatty liver disease (MASLD), the metabolic interactions between the gut and liver are particularly significant. Statistics indicate that approximately 38% of adults and 7% to 14% of children and adolescents worldwide currently suffer from MASLD, with adult prevalence projected to exceed 55% by 2040 [16]. This high prevalence underscores the critical role of gut dysbiosis in driving systemic metabolic disorders. According to the Remote Sensing and Signaling Theory, the gut microbiota constructs an extensive signaling network by generating small-molecule metabolites (such as nutrients, antioxidants, and uremic toxins) that traverse transporters, directly influencing the homeostasis of distant organs like the heart and kidneys [17].

At the pathophysiological level, gut microbiota metabolites can directly interfere with host mitochondrial function and energy metabolism. Research indicates that indole-3-sulfonic acid (IS), a key metabolic toxin produced by Escherichia coli in the gut via the tryptophanase (TnaA) pathway, disrupts cardiac mitochondrial function by activating the AHR-CYP1B1 axis. This process induces cardiomyocyte apoptosis and accelerates the progression of heart failure [18]. This microbiota-driven metabolic disorder extends beyond the heart, exacerbating cardiovascular risk in chronic kidney disease (CKD) patients through complex “gut–kidney–heart” crosstalk mechanisms. Conversely, beneficial microbiota and their metabolites demonstrate significant protective effects. For instance, *Faecalibacterium prausnitzii* is markedly reduced in CKD patients. Supplementing this microorganism activates the renal GPR-43 signaling pathway via its metabolite butyrate, thereby suppressing renal inflammation, improving intestinal barrier integrity, and restoring systemic metabolic homeostasis [19].

Furthermore, the management of complications associated with metabolic disorders is highly dependent on interventions targeting this multi-organ metabolic network. Gout, a prevalent form of inflammatory arthritis, exhibits an incidence rate ranging from 0.58‰ to 2.89‰, with global prevalence varying from <1% to 6.8% [20]. Gout is closely associated with metabolic syndrome, chronic kidney disease (CKD), and cardiovascular disease. Treatment strategies are increasingly shifting towards medications such as SGLT2 inhibitors, which simultaneously improve cardiac, renal, and metabolic outcomes. This shift indirectly underscores the central role of metabolic regulation in the management of systemic diseases [20,21].

Therefore, to bridge the current knowledge gaps, this comprehensive review aims to systematically decode the complex “gut–fat–heart” axis from a holistic metabolic perspective. The specific objectives are threefold: (1) to delineate the physiological communication networks by which the gut microbiota remotely regulates host lipid homeostasis via bile acid signaling, short-chain fatty acids (SCFAs), and neuroendocrine reflexes; (2) to map the pathological mechanisms driven by microbial dysbiosis and specific metabolites—such as trimethylamine N-oxide (TMAO), lipopolysaccharides (LPS), and bacterial outer membrane vesicles (OMVs)—that trigger ectopic lipotoxicity, vascular inflammation, and structural cardiac remodeling; and (3) to critically evaluate emerging microbiome-targeted clinical translation strategies, ranging from next-generation probiotics and molecularly targeted drugs to phytochemical interventions and bariatric surgery. By synthesizing these multifaceted insights, this manuscript seeks to provide a definitive molecular framework and highlight actionable therapeutic targets for the integrated management of cardiovascular–kidney–metabolic (CKM) syndrome.

## 2. Physiological Regulation: How the Gut “Remotely Controls” Lipid Metabolism

Before delineating the specific pathophysiological axes driven by dysbiosis, it is essential to establish the desired profile of bacterial communities and their metabolites that actively sustain cardiovascular and metabolic health. In a resilient superorganism, a diverse microbiome—enriched with commensal keystone taxa such as *Akkermansia muciniphila*, *Faecalibacterium prausnitzii*, and *Bifidobacterium* species—operates as a synchronized metabolic engine. The optimal microbial metabolomic profile is primarily characterized by the robust production of short-chain fatty acids (SCFAs), particularly butyrate and propionate. These SCFAs are indispensable for maintaining tight junction integrity, fueling colonocytes, and exerting systemic anti-inflammatory effects via host G-protein coupled receptors. Concurrently, a healthy microbial landscape maintains a balanced turnover of primary to secondary bile acids (e.g., lithocholic and ursodeoxycholic acids) that optimally engage host nuclear receptors, such as FXR and TGR5, without exceeding toxicity thresholds. Furthermore, a homeostatic microbiome efficiently converts dietary tryptophan into cardioprotective indole derivatives, such as indole-3-propionic acid (IPA), which actively scavenge free radicals and preserve endothelial function. Together, this integrated chemical vocabulary—abundant SCFAs, balanced bile acid derivatives, and protective indoles—establishes a functional baseline that continuously suppresses low-grade inflammation, facilitates efficient postprandial lipid clearance, and fortifies the gut–fat–heart axis against metabolic stress.

### 2.1. Bile Acid–FXR/FGF15/19 Axis: Negative Feedback Regulation of Lipid Synthesis

**Figure 2:**
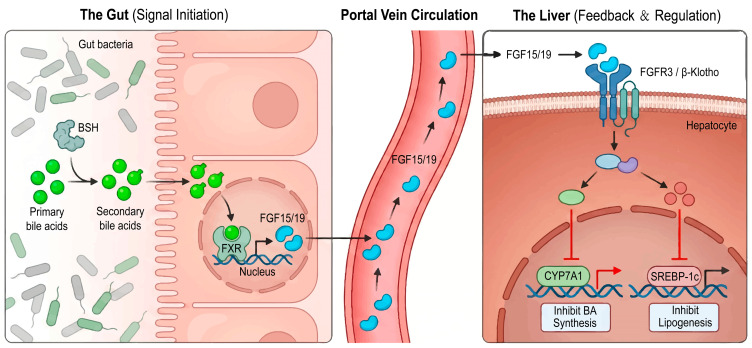
Physiological Mechanism Map—Bile Acid–FXR Enterohepatic Circulation & Lipid Homeostasis. BSH, bile salt hydrolase; FXR, farnesoid X receptor; FGF15/19, fibroblast growth factor 15/19; FGFR4, fibroblast growth factor receptor 4; CYP7A1, cholesterol 7 alpha-hydroxylase; SREBP-1c, sterol regulatory element-binding protein 1c.

The gut microbiota constitutes the primary line of defence in regulating host lipid metabolism through the modification of the bile acid (BA) pool via bile salt hydrolase (BSH). BSH activity determines the rate of conversion from conjugated to unconjugated bile acids, a process that directly reshapes the spectrum of signalling molecules within the intestine. Research indicates that the colonisation of specific probiotic strains (such as *Lactobacillus acidophilus* and *Lactobacillus plantarum*) or commensal bacteria (such as *Bacteroides eggerthii*) significantly enhance choline glycine hydrolase activity within the gut. This promotes the dissociation of conjugated bile acids into free bile acids (e.g., CDCA, CA), which are subsequently converted into secondary bile acids [22,23,24]. This metabolic conversion not only alters the hydrophilic–lipophilic balance within the bile acid pool but, more importantly, generates high-affinity ligands for the farnesoid X receptor (FXR), thereby initiating cross-organ metabolic signalling pathways [23,25] (Figure 2).

Activation of intestinal FXR triggers the classic FGF15/19-CYP7A1 negative feedback loop, a key mechanism for maintaining bile acid homeostasis and suppressing excessive lipid production. When FXR in the intestine is activated by microbially modified bile acids such as CDCA, it induces the secretion of fibroblast growth factor 15/19 (FGF15 in rodents, FGF19 in humans). FGF15/19 travels via the portal vein to the liver, where it binds to the FGFR4/β-Klotho receptor complex, thereby inhibiting the transcriptional expression of cholesterol 7α-hydroxylase (CYP7A1) and consequently reducing de novo bile acid synthesis [24,25,26]. Activation of this axis is crucial for preventing toxic accumulation of bile acids within the liver, whilst simultaneously limiting exogenous lipid intake at its source by regulating the expression of intestinal lipid absorption transporters such as ASBT and NPC1L1 [22,26].

At the molecular regulatory level of de novo lipid synthesis (DNL) in the liver, the FXR-SHP-SREBP-1c axis functions as a master switch. Exposure to environmental pollutants (such as Microcystin-LR) suppresses hepatic FXR expression, thereby releasing inhibition on sterol regulatory element-binding protein 1 (SREBP-1) and triggering uncontrolled triglyceride synthesis [26]. Conversely, activating FXR via specific traditional Chinese medicine formulations—such as Simiaowan (also referred to as Si Miao Fang, a classic four-herb formulation that standardizes the delivery of metabolic modulators including berberine and atractylodin)—or probiotic metabolites upregulates small heterodimer partner (SHP) expression. Acting as a transcriptional repressor, SHP directly blocks the transcriptional activity of SREBP-1c and its downstream lipogenic genes (e.g., Fas, Acc), thereby significantly reducing hepatic lipid accumulation [27]. Furthermore, certain natural products (e.g., rhodiola glycosides) have been demonstrated to modulate gut microbiota composition, thereby reducing levels of inhibitory bile acids (e.g., T-β-MCA). This relieves their suppression of FXR/TGR5, subsequently downregulating SREBP-1c and activating fatty acid oxidation pathways [28]. In summary, the gut microbiota remotely modulates the FXR-FGF15/19 axis and hepatic FXR-SREBP-1c pathway by modifying bile acid signalling molecules via BSH enzymes, constituting a sophisticated physiological network defending against hyperlipidaemia and fatty liver disease.

### 2.2. Short-Chain Fatty Acids: Energy Balance and Substrate Competition

Among short-chain fatty acids (SCFAs) derived from gut microbiota, acetate exhibits a pronounced “double-edged sword” metabolic property. On the one hand, acetate serves as a key substrate for hepatic lipid synthesis. Research indicates that via the acetyl-CoA synthase 2 (ACSS2)-mediated pathway, acetate can be converted into acetyl-CoA, subsequently participating in de novo lipogenesis (DNL) of fatty acids and cholesterol [29,30]. Particularly when acetyl-CoA lyase (ACLY) is inhibited, the liver undergoes metabolic reprogramming, utilising acetate as the primary carbon source to sustain lipid synthesis. This ACSS2-dependent metabolic flux may even exacerbate fatty liver progression [30,31]. Moreover, within the tumour microenvironment, increased acetate uptake has been demonstrated to promote tumour growth by supplying acetyl-CoA to support lipid biosynthesis and histone acetylation in tumour cells [32,33]. Conversely, acetate also performs crucial metabolic regulatory functions. It can inhibit lipid peroxidation and ferroptosis by activating the hepatic AMPK/SIRT1/PGC-1α signalling axis [34], and block the progression from non-alcoholic fatty liver disease to hepatocellular carcinoma by inhibiting pro-inflammatory signalling pathways (such as IL-6/JAK1/STAT3) via binding to G protein-coupled receptor 43 (GPR43/FFAR2) [35]. Further studies indicate that acetate can inhibit fat deposition via the hepatic AMPK-PPARα axis and shift metabolism towards fatty acid oxidation [36,37]. Consequently, acetate’s ultimate effect on lipid metabolism depends on the balance between its roles as a synthetic substrate and a signalling molecule.

Unlike the substrate characteristics of acetate, propionate exhibits a more pronounced protective effect in regulating cholesterol homeostasis. Systematic evaluations indicate that combined intervention with acetate and propionate demonstrates optimal efficacy in reducing total cholesterol and triglycerides [38]. Its mechanism involves complex receptor-mediated networks. For instance, G protein-coupled receptor 41 (GPR41/FFAR3) serves as a key intestinal chemosensor; its absence leads to altered intestinal motility and disrupted bile acid metabolism, subsequently affecting lipid absorption by downregulating expression of the ileal cholesterol transporter Npc1l1 [39]. Furthermore, propionic acid derived from specific gut symbionts (e.g., *Akkermansia muciniphila*) has been demonstrated to effectively ameliorate metabolic dysregulation and suppress endoplasmic reticulum stress [40]. In studies of high-yielding dairy cows, elevated ruminal propionic acid levels were also closely associated with optimised lipid metabolism modules and a positive correlation with milk yield (energy output) [41], suggesting propionic acid plays a central regulatory role in energy allocation and lipid homeostasis.

Butyrate primarily exerts its effects through epigenetic modifications and immunometabolic reprogramming to finely regulate lipid metabolism. As a potent histone deacetylase (HDAC) inhibitor, butyrate reshapes the cellular epigenetic landscape. Research indicates that butyrate enhances histone H3K27 acetylation levels in the promoter regions of PPARD and fatty acid oxidation (FAO)-related genes by inhibiting HDAC3 function. This drives the fatty acid oxidation programme in myeloid-derived suppressor cells (MDSCs), thereby restoring immune and metabolic homeostasis [42]. Furthermore, butyrate induces chromatin remodelling via STAT family transcription factors, regulating chemokine expression to improve the microenvironment [43]. In diet-induced obesity and fatty liver models, butyrate not only reduces lipid accumulation by activating the AMPK pathway [44], but has also been demonstrated to be the most effective SCFA intervention for lowering fasting blood glucose [38]. Specific probiotic interventions (e.g., *Lactiplantibacillus plantarum*) significantly elevate intestinal butyrate levels, thereby exerting anti-obesity and lipid-lowering effects through remodelling gut–liver axis signalling (such as restoring FXR signalling and regulating bile acid metabolism) [38].

In summary, the role of SCFAs in lipid metabolism is not a simple additive effect, but rather a finely tuned interplay between substrates and signalling pathways. Acetate provides the carbon skeleton for lipid synthesis while simultaneously inhibiting lipid accumulation through signalling pathways [29]. Propionate restricts cholesterol absorption via receptor mechanisms [39], whereas butyrate reprograms lipid oxidation metabolism at the transcriptional level through epigenetic mechanisms [42]. This metabolic flexibility implies that precise interventions targeting the gut microbiota must comprehensively consider the ratio of these three SCFAs alongside the host’s metabolic state.

### 2.3. Gut–Brain–Liver Axis: Lipid Regulation of the Vagus Nerve

The interaction between gut microbiota and host lipid metabolism relies not only on the systemic circulation of metabolites but also on a more rapid ‘microbiota–gut–brain–liver’ neuro-reflex pathway. Research confirms that specific gut bacteria and their metabolic products can act as ‘neuroactive signals’, directly activating vagal afferent fibres to transmit nutritional and metabolic states from the gut to the central nervous system. This subsequently regulates systemic energy balance and hepatic physiological functions. This neurosensory mechanism exhibits high specificity. For instance, neuropod cells within the intestinal epithelium can detect bacterial flagellin via Toll-like receptor 5 (TLR5), rapidly releasing peptide YY (PYY) to activate vagal ganglion neurons expressing NPY2R. This directly suppresses appetite and feeding behaviour independently of immune or metabolic alterations [45]. Furthermore, tryptophan metabolites (indole derivatives) produced by gut microbiota such as Edwardsiella tarda activate TRPA1 channels on enteroendocrine cells, inducing serotonin (5-HT) secretion. This subsequently excites vagal sensory ganglia and cholinergic enteric neurons, forming a ‘microbiota–gut–vagus’ signalling cascade [46].

In terms of lipid-specific regulation, the gut microbiota plays a crucial role in the lipid-sensing mechanisms within the small intestine. Dietary lipids typically activate the gut–brain axis to regulate energy homeostasis, whereas dysbiosis induced by high-fat diets disrupts this mechanism. Research indicates that interventions with prebiotics (such as fructooligosaccharides) or specific bacterial strains (e.g., *Bifidobacterium pseudolongum*) can restore small intestinal microbiota structure, enhance CD36 expression in the small intestine and portal vein GLP-1 levels, thereby reinstating lipid-sensing capacity in hindbrain neurons. This suppresses excessive feeding and ameliorates obesity-associated lipid metabolism disorders [47]. This microbe-mediated neural signalling can even reshape hypothalamic activity patterns. For instance, differential fermentation of complex carbohydrates (such as fructans) by gut bacteria can selectively activate the hypothalamic arcuate nucleus, thereby regulating host food selection and intake by sensing energy extraction efficiency [48].

Once afferent signals are integrated within the nucleus tractus solitarius (NTS), the efferent vagal motor fibers orchestrate hepatic lipid and immune homeostasis primarily through the cholinergic anti-inflammatory pathway (CAP). Recent high-resolution 3-D imaging has confirmed direct parasympathetic (VAChT+) innervation within the human liver [49]. Upon vagal stimulation, efferent terminals release acetylcholine (ACh), which specifically binds to the α7 nicotinic acetylcholine receptor (α7nAChR) expressed on hepatic macrophages and Kupffer cells. Mechanistically, α7nAChR activation triggers the JAK2/STAT3 signaling cascade, which subsequently profoundly inhibits the NF-κB pathway [50,51,52]. This targeted suppression not only blunts the transcription of pro-inflammatory cytokines but also directly downregulates lipogenic gene expression and modulates cholesterol metabolism [51]. Conversely, the interruption of this liver–brain axis via vagotomy significantly shifts the hepatic metabolic landscape, increasing glycolytic and fatty acid biosynthesis while decreasing β-oxidation, thus facilitating ectopic lipid accumulation [53]. Consequently, this efferent neuro-immune reflex establishes a precise molecular circuit by which gut microbial sensing is translated into hepatic metabolic control and inflammation resolution [54].

### 2.4. Gut Hormones: GLP-1 and Chylomicron Assembly

**Figure 3:**
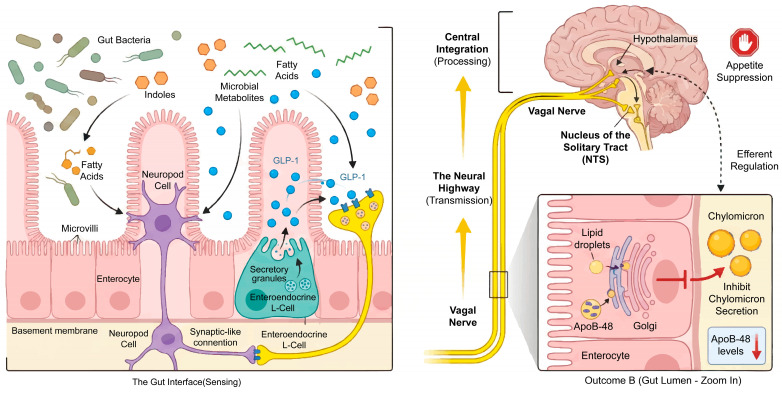
Neuro-Endocrine Map—Synergistic Regulation by Vagal Reflex and GLP-1. GLP-1, glucagon-like peptide-1; ApoB-48, apolipoprotein B-48; NTS, nucleus of the solitary tract.

Intestinal endocrine cells (particularly L cells), acting as nutrient sensors, not only secrete hormones to regulate blood glucose but also play a pivotal role in overall energy homeostasis by modulating intestinal lipid absorption and metabolism. The gut microbiota and its metabolites play a significant role in this process, capable of directly or indirectly stimulating the secretion of GLP-1 (glucagon-like peptide-1). For instance, dietary intake of prebiotics such as fructooligosaccharides can rapidly alter small intestinal microbiota composition, notably increasing *Bifidobacterium* abundance. This subsequently enhances lipid sensing mechanisms in the small intestine, thereby promoting GLP-1 secretion [47,55]. Moreover, specific gut bacteria (e.g., *Bacteroides thetaiotaomicron*) can upregulate intestinal GLP-1 expression, potentially by restoring downregulation of FGF15, thereby ameliorating alcohol-induced hepatic steatosis [56]. Notably, certain bacterial metabolites, such as short-chain fatty acids (SCFAs) and indole derivatives, have also been demonstrated to stimulate gut hormone secretion by activating specific receptors on enteroendocrine cells (e.g., TRPA1) [46,47,55] (Figure 3). These findings reveal the pivotal role of the ‘microbiota–metabolite–L cell’ axis in regulating GLP-1 secretion.

The regulatory role of GLP-1 in lipid metabolism extends beyond its well-known insulin-secretagogue effect, exerting direct action upon intestinal lipid processing, particularly by inhibiting the assembly and secretion of postprandial chylomicrons. Mechanistic studies indicate that GLP-1 receptor agonists (GLP-1RAs) significantly reduce postprandial triglyceride (TG) and apolipoprotein B-48 (ApoB-48) levels, with ApoB-48 serving as the core structural protein of chylomicrons [57,58]. This inhibitory effect is partially mediated by downregulating key genes involved in intestinal lipoprotein metabolism (such as CD36, FATP-2, FATP-4, and APOA-4), thereby reducing intestinal lipid absorption and transport [57,58,59]. Moreover, activation of the GLP-1 signalling pathway is finely regulated via the sympathetic-enteric L-cell unit. Sympathetic activation limits GLP-1 release, whereas blocking this inhibitory signal enhances postprandial GLP-1 secretion, thereby improving glucose and lipid metabolism [60]. In clinical trials, GLP-1RA therapy not only reduced fasting and postprandial triglyceride levels but also accelerated the catabolism of triglyceride-rich lipoproteins (TRLs). This holds significant implications for reducing cardiovascular risk in patients with type 2 diabetes mellitus [58].

More intriguingly, dual agonists of GLP-1 and GIP (glucose-dependent insulinotropic polypeptide), such as Tirzepatide, demonstrate stronger lipid-regulating capabilities than single GLP-1 receptor agonists. These dual agonists not only more effectively reduce body weight and blood glucose but also synergistically regulate adipose tissue metabolism: enhancing glucose and lipid clearance during feeding while promoting lipolysis during fasting [61,62]. This synergistic action on multiple hormone receptors mimics the metabolic remodeling effects following bariatric surgery (e.g., Roux-en-Y gastric bypass), which improves systemic metabolic health through supraphysiological levels of gut hormone secretion [61,63]. Furthermore, novel dietary intervention strategies—such as physically blocking intestinal lipid absorption via interface protein fiber polymorphisms—have been shown to significantly reduce ApoB-48 and chylomicron size, with effects complementary to GLP-1 pathway regulation [59]. In summary, GLP-1 and its associated signaling pathways constitute a critical component of the gut’s “remote control” over systemic lipid metabolism by directly inhibiting intestinal ApoB-48 synthesis and chylomicron secretion.

### 2.5. Intestinal Epithelial CD36 and the Physical Barrier

As the key “gateway” for intestinal lipid absorption, the Cluster of Differentiation 36 (CD 36) transporter on the brush border of intestinal epithelial cells, together with the intestinal physical barrier, determines the efficiency of fatty acid uptake. The gut microbiota and its metabolites exert precise regulatory effects on this process. By altering CD36 expression levels or modification states and modulating the integrity of the physical barrier, they directly influence the host’s lipid metabolic homeostasis.

The gut microbiota exhibits bidirectional regulatory capacity over CD36 expression in intestinal epithelium, thereby directly influencing the host’s fatty acid absorption efficiency. On one hand, specific bacterial strains can promote CD36 expression to enhance lipid absorption. For example, Lactobacillus johnsonii isolated from obese models has been shown to promote intestinal fatty acid absorption and lipid deposition. This mechanism involves secreting the metabolite (R)-leucic acid, which targets and upregulates CD36 expression in intestinal epithelial cells [64]. Similarly, supplementation with Bacteroides fragilis induces dysbiosis, significantly upregulating CD36 mRNA levels in small intestinal tissue, thereby exacerbating metabolic dysfunction and atherosclerosis [65]. Conversely, probiotics and specific dietary interventions can suppress CD36 expression. For example, Lactobacillus paragasseri SBT2055 exerts anti-obesity effects by reducing mRNA levels of CD36, FABP1/2, and ApoB-48 in the small intestine, thereby increasing fecal lipid excretion [66]. Furthermore, isoxanthohumol intake specifically promotes *Akkermansia muciniphila* abundance, significantly reducing jejunal CD36 expression and inhibiting intestinal lipid absorption [67].

The regulation of CD36 by the gut microbiota involves complex transcriptional mechanisms and is closely associated with the integrity of the intestinal physical barrier. Research has demonstrated that konjac glucomannan (KGM) and its degradation products suppress downstream transcription of CD36 and FABP1 by downregulating the expression of HDAC3 and nuclear factor-κB-like interleukin-3-related protein (NFLI3) in the intestine. SREBP1, and FABP1. This process is accompanied by enhanced intestinal barrier function and increased short-chain fatty acid (SCFA) production [68]. Disruption of the physical barrier frequently coincides with abnormal elevation of CD36. For instance, ingestion of trans fatty acids or microplastics not only induces intestinal inflammation and barrier impairment but also significantly induces overexpression of CD36 in the small intestine, promoting excessive lipid uptake and metabolic dysregulation [69,70].

Based on an understanding of CD36 and physical barrier mechanisms, novel biomaterials have been engineered to physically impede lipid absorption. For instance, hybrid hydrogels formed by the self-assembly of flavonoids and protein amyloid fibrils not only enhance the expression of intestinal tight junction proteins (such as Claudin-1) but also significantly reverse the overexpression of host intestinal lipid absorption genes (CD36 and NFIL3) induced by a high-fat diet [71]. Furthermore, engineered interfacial protein fibril polymorphisms have been demonstrated to inhibit lipolysis by forming dense interfacial layers, concurrently downregulating intestinal CD36 and transporter (FATP-2/4) expression, thereby reducing chylomicron assembly and secretion [59]. These findings indicate that modulating the ‘microbiota-CD36-physical barrier’ axis can effectively control the bioavailability of dietary fat.

## 3. Pathological Mechanism: Lipid Toxicity Induced by Bacterial Metabolites

### 3.1. TMAO: Reverse Transport Blockage and Foam Cell Formation

**Figure 4:**
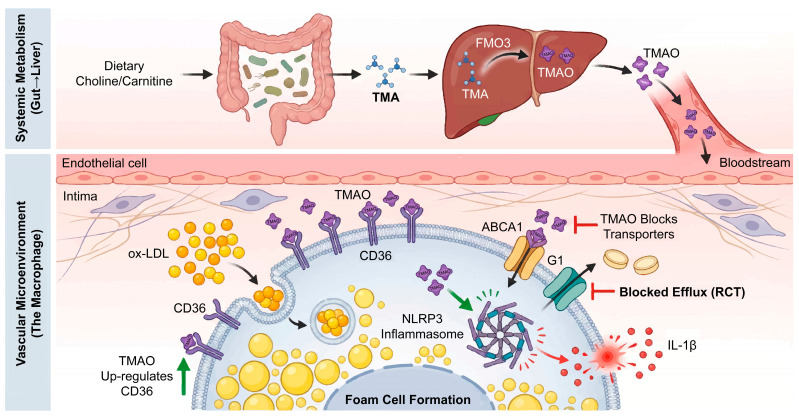
Core Pathology A—TMAO-Induced “Foam Cell” Formation and Vascular Inflammation. TMA, trimethylamine; TMAO, trimethylamine N-oxide; FMO3, flavin-containing monooxygenase 3; ox-LDL, oxidized low-density lipoprotein; ABCA1/ABCG1, ATP-binding cassette transporter A1/G1; NLRP3, NLR family pyrin domain containing 3; IL-1β, interleukin-1β.

Trimethylamine N-oxide (TMAO), as a key gut microbiota-derived metabolite, exerts significant lipotoxic effects in the pathological progression of atherosclerosis. Its core mechanisms involve disruption of cholesterol homeostasis and accelerated macrophage foaming. Research indicates that TMAO significantly impedes reverse cholesterol transport (RCT), primarily through its inhibition of key lipid efflux transporters [72,73]. Specifically, TMAO exposure downregulates the expression of ABCA1 and ABCG1 in macrophages, a process typically accompanied by suppression of the transcriptional activity of upstream nuclear receptors PPARγ and LXRα [74,75]. Since ABCA1 and ABCG1 are responsible for effluxing intracellular cholesterol to ApoA-I and HDL, their impaired function prevents efficient lipid clearance. This leads to excessive lipid accumulation within macrophages and accelerates foam cell formation [75]. Furthermore, elevated plasma TMAO levels resulting from PSRC1 deficiency have been demonstrated to further impair hepatic cholesterol transport capacity, exacerbating lipid deposition within plaques [76] (Figure 4).

While inhibiting lipid efflux, TMAO also promotes lipid uptake by upregulating scavenger receptors and elicits robust inflammatory responses. Elevated TMAO levels correlate closely with increased expression of macrophage scavenger receptors such as SRA and CD36, enhancing cellular phagocytosis of oxidized low-density lipoprotein (ox-LDL) [77,78]. Beyond promoting macrophage polarization toward the proinflammatory M1 phenotype, TMAO induces the release of proinflammatory cytokines like IL-1β, IL-6, and TNF-α by activating the NF-κB signaling pathway and NLRP3 inflammasome [79,80,81]. This immunometabolic dysregulation not only sustains the microenvironment for foam cells but also increases atherosclerotic plaque instability by degrading the extracellular matrix (ECM) and promoting apoptosis [82,83].

At the molecular mechanism level, endoplasmic reticulum (ER) stress and its associated PERK signaling pathway are key drivers of TMAO-induced lipid toxicity and cellular damage. TMAO specifically activates the PERK (Protein Kinase R-like Endoplasmic Reticulum Kinase)/ATF-4 signaling axis, triggering explosive production of reactive oxygen species (ROS) within cells [84]. Studies reveal that in vascular smooth muscle cells and other cellular models, TMAO-mediated PERK pathway activation further triggers downstream cascades such as CaMKII/PLCβ3, leading to intracellular calcium homeostasis disruption and oxidative stress damage [85]. This sustained oxidative stress mediated by the PERK pathway forms a positive feedback loop with NLRP3 inflammasome activation, jointly exacerbating vascular remodeling and pathological fibrosis, ultimately driving the progression of atherosclerotic lesions [84,86]. However, the universal pathogenicity of TMAO is complicated by the “seafood paradox”: despite marine fish being rich sources of TMAO precursors, their consumption strongly correlates with cardioprotection. Such contradictory findings suggest that in certain clinical contexts, circulating TMAO might serve as a biomarker of underlying metabolic or renal dysfunction rather than a direct pathogenic driver.

### 3.2. LPS and “Dysfunctional HDL”

Disruption of the intestinal barrier function leading to lipopolysaccharide (LPS) translocation, known as metabolic endotoxemia, is a key pathological mechanism triggering high-density lipoprotein (HDL) dysfunction and chronic inflammation. In metabolic syndrome, sepsis, and cardiovascular disease, dysbiosis and intestinal permeability (leaky gut) allow LPS from Gram-negative bacteria to enter the bloodstream, triggering systemic inflammation [84,87]. Studies indicate that LPS activates the TLR4 signaling pathway, inducing the liver to produce large amounts of serum amyloid A (SAA) [88]. SAA, an acute-phase reactant protein, displaces apolipoprotein A-I (ApoA-I)—the primary structural protein on HDL particles—during inflammation, leading to significant proteomic remodeling of HDL [89].

These SAA-enriched HDL particles (SAA-HDL) not only lose their original anti-inflammatory and antioxidant functions but may even transform into pro-inflammatory factors. Proteomic analysis revealed significantly reduced ApoA-I levels in HDL from patients with sepsis-induced acute respiratory distress syndrome (ARDS), alongside elevated SAA and pro-SFTPB (pro-surfactin B precursor) levels. This compositional shift directly promotes macrophage polarization toward the M1 pro-inflammatory phenotype, exacerbating tissue injury [89]. Furthermore, SAA-HDL is readily captured by proteoglycans on adipose tissue and macrophage surfaces, such as versican and biglycan, thereby losing its ability to inhibit inflammatory signaling and clear cholesterol. This process further impairs HDL-mediated cholesterol reverse transport and vascular protective effects [88].

The formation of dysfunctional HDL is also accompanied by a decline in its antioxidant enzyme activity, particularly the loss of phospholipase A1 (PON1) activity. PON1 typically binds to HDL and is responsible for hydrolyzing oxidized lipids. However, under inflammatory and oxidative stress conditions (such as diabetes and acute coronary syndrome), HDL undergoes oxidative modification (oxHDL) and glycation, impairing PON1 activity and its ability to effectively counteract LDL oxidation [90,91]. This oxidatively modified HDL not only fails to promote cholesterol efflux but also correlates positively with high-risk coronary plaque characteristics such as necrotic cores and fibrous fat loading [92]. Thus, the LPS-induced HDL proteomic remodeling, particularly the displacement of ApoA-I by SAA, represents a core mechanism transforming HDL from a “vascular guardian” into a “dysfunctional particle,” a process that fuels the progression of atherosclerosis and metabolic diseases.

### 3.3. Aromatic Hydrocarbon Receptor (AhR) Ligands: The Vascular Toxicity of Tryptophan Metabolites

The gut microbiota metabolizes dietary tryptophan into multiple indole derivatives, which act as endogenous ligands for the aryl hydrocarbon receptor (AhR) and exert complex bidirectional regulatory effects on cardiovascular health. On one hand, uremic toxins such as indole sulfate (IS), which accumulate in CKD, exhibit significant vasotoxicity. IS activates AhR in endothelial cells, upregulating cytochrome P450 family members (e.g., CYP1A1) to induce oxidative stress and inflammatory responses [93,94]. Specifically, activation of the IS-AhR axis promotes endothelial expression of vascular cell adhesion molecule-1 (VCAM-1) and monocyte chemotactic protein-1 (MCP-1). This not only recruits monocytes to adhere to the vascular wall but also exacerbates endothelial inflammation via the non-classical TGF-β pathway, creating conditions conducive to lipid deposition and atherosclerotic plaque formation [93,95]. Furthermore, IS can impair endothelial cell luminal formation and proliferation capabilities through AhR-dependent mechanisms, inhibiting angiogenesis and thereby exacerbating ischemic injury [96].

However, not all AhR ligands are detrimental. Another tryptophan metabolite derived from the gut microbiota—indole-3-propionic acid (IPA)—exhibits significant cardioprotective effects in heart failure with preserved ejection fraction (HFpEF). IPA binds AhR to promote mitochondrial SIRT3 expression, restore NAD+ levels, and thereby improve myocardial energy metabolism and diastolic function [97]. Similarly, indole-3-lactic acid (ILA) activates AhR to enhance endothelial cell proliferation and migration via the PI3K/AKT pathway, alleviating preeclampsia-associated vascular dysfunction [98].

This differential response indicates that the consequences of AhR pathway activation are highly dependent on the specific ligand type and its pathophysiological context. For instance, in atherosclerosis models, dendritic cell overexpression of IDO1 increases local kynurenine levels, amplifying regulatory T cell (Treg) modulation via AhR and thereby reducing vascular inflammation and plaque burden [99]; whereas IS tends to induce vascular smooth muscle cell senescence and calcification via AhR, exacerbating vascular stiffening [100]. Thus, gut microbiota-derived indole metabolites maintain a delicate equilibrium between sustaining vascular homeostasis and promoting pathological remodelling through the AhR hub, with their ultimate effects contingent upon metabolite type, concentration, and the microenvironment of target cells.

### 3.4. Branched-Chain Amino Acids: Insulin Resistance and Lipid Accumulation

Gut microbiota-mediated dysregulation of branched-chain amino acid (BCAA) metabolism constitutes a pivotal link connecting insulin resistance to ectopic lipid deposition. Specific gut symbionts (such as Prevotella copri and Bacteroides vulgatus) possess active BCAA biosynthetic capabilities, with increased abundance correlating closely to elevated serum BCAA levels and insulin resistance [101,102]. However, the metabolic kinetics and downstream pathological effects of the three BCAAs diverge significantly [103].

Leucine primarily functions as a potent nutrient signal rather than a mere substrate, exerting its effects through a highly specific intracellular sensing apparatus. The cytosolic leucine sensor Sestrin2 physically binds leucine with high affinity through a specific binding pocket that coordinates its charged functional groups [104]. Under leucine-deprived conditions, Sestrin2 maintains a highly flexible conformation and acts as a direct inhibitor of the GATOR2 complex [105]. However, upon leucine binding, Sestrin2 undergoes an allosteric conformational locking that triggers its dissociation from GATOR2 [106]. This dissociation relieves the structural inhibition on GATOR2, which subsequently suppresses the GAP activity of the GATOR1 complex [105,106]. Consequently, Rag GTPases become activated and physically recruit the mTORC1 complex to the lysosomal surface for full activation [106]. In the liver, this Sestrin2-mTORC1 sensing mechanism is highly spatially organized (zonated) and critically regulates hepatic lipid metabolism and FGF21 production in response to dietary leucine [107]. This persistent, leucine-driven overactivation of the mTORC1 signaling axis ultimately leads to the hyperphosphorylation of insulin receptor substrates (IRS-1), establishing a profound state of peripheral insulin resistance.

Conversely, the kinetics of valine and isoleucine are intrinsically linked to lipid toxicity. For instance, the excessive catabolism of valine generates 3-hydroxyisobutyrate (3-HIB), a paracrine metabolite that promotes trans-endothelial fatty acid transport [108], directly driving ectopic free fatty acid (FFA) flux and lipid accumulation in target organs such as the liver and skeletal muscle [109]. The systemic accumulation of these BCAAs is further exacerbated by a microbiota-induced bottleneck in host catabolism. Following the initial transamination by branched-chain amino acid transaminase (BCAT), the rate-limiting step of BCAA degradation is governed by the branched-chain α-keto acid dehydrogenase (BCKDH) complex. The enzymatic activity of BCKDH is tightly regulated by a phosphorylation toggle: it is inactivated by BCKDH kinase (BCKDK) and activated by the phosphatase PPM1K. In states of microbiota dysbiosis and associated metabolic inflammation, host hepatic and adipose tissues exhibit a marked upregulation of BCKDK and a concomitant suppression of PPM1K. This regulatory imbalance results in the persistent hyperphosphorylation and functional inactivation of the BCKDH complex [110].

This critical enzymatic blockade prevents the oxidative decarboxylation of branched-chain keto acids (BCKAs), leading to the systemic pooling of BCAAs. Conversely, promoting the catabolism of BCAAs by bypassing this bottleneck can effectively improve metabolic health. Gut microbiota (e.g., *Lactobacillus reuteri*) and specific metabolites can upregulate host BCAT2 expression and restore BCKDH activity via epigenetic mechanisms, accelerating BCAA degradation and improving insulin sensitivity [111]. Furthermore, mitochondrial BCAA catabolism in brown adipose tissue (BAT) serves as an independent mechanism regulating systemic metabolic health; impaired flux through the BCKDH complex in BAT leads to increased oxidative stress and systemic metabolic dysfunction [112], whereas promoting this catabolic bypass efficiently clears systemic BCAAs [113]. Dietary interventions (such as resistant starch) can also reduce serum BCAA levels by reshaping the gut microbiota, thereby alleviating hepatic fat deposition driven by this metabolic axis [114].

### 3.5. Bacterial Outer Membrane Vesicles and Sphingolipids

Bacterial outer membrane vesicles (OMVs) serve as pivotal mediators for cross-barrier communication between gut microbiota and the host, emerging as novel signalling hubs linking the gut microbiome to cardiovascular pathology. Research confirms that OMVs are not confined to the intestinal microenvironment but possess the capacity to traverse biological barriers. In vivo tracing techniques reveal that OMVs released by gut bacteria (such as Escherichia coli) can breach the intestinal barrier to enter the bloodstream, subsequently distributing extensively to distant organs including the liver, kidneys, and heart [115]. In metabolic dysfunction-related diseases, OMVs derived from gut symbionts (e.g., *Alistipes timonensis*) have been found to carry specific lipid components into the host circulatory system, significantly modulating host plasma lipidomics profiles—such as elevating phosphatidic acid levels while reducing triglycerides—demonstrating OMVs as effective carriers for microbial lipids regulating host systemic lipid metabolism [116].

At the molecular level, the biological efficacy of bacterial outer membrane vesicles (OMVs) is strictly governed by their cellular internalization mechanisms. OMVs are primarily internalized into host cells via endocytic pathways, a process facilitated by specific host factors such as the DYNLL2-PAK1 axis or AAK1 [117,118]. Once internalized, OMVs bypass extracellular immune surveillance, traffic through early endosomes to lysosomes, and release their enriched cargo—crucially including lipopolysaccharides (LPS)—directly into the host cytosol [119,120]. Cytosolic LPS binds directly to the non-canonical inflammasome receptors Caspase-4 (in humans) or Caspase-11 (in mice) [121,122]. This interaction, which can be further amplified by host cytosolic sensors like Galectin-3 [122], triggers caspase oligomerization and activation, leading to gasdermin D (GSDMD) cleavage and subsequent pyroptosis [117,123]. Concurrently, specific OMV components can induce profound mitochondrial dysfunction, promoting massive reactive oxygen species (ROS) generation and initiating a synergistic crosstalk between pyroptotic and apoptotic/cuproptotic cell death pathways [124]. This explosive cell death and the consequent release of damage-associated molecular patterns (DAMPs) lock the local tissue environment into an irreversible trajectory of severe inflammation and immune-mediated damage.

**Figure 5:**
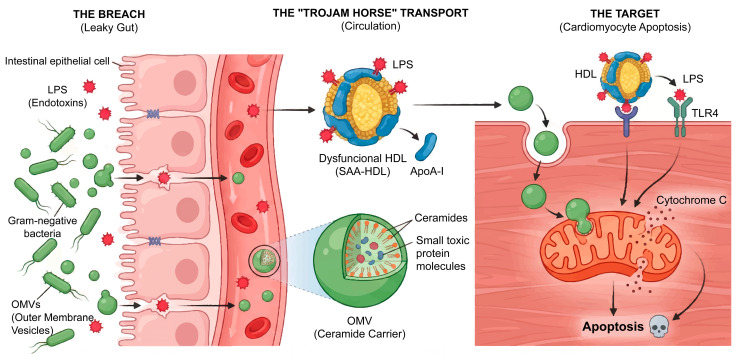
Core Pathology B—The “Trojan Horse” Invasion: Bacterial OMVs and LPS hijacking the Heart. LPS, lipopolysaccharide; HDL, high-density lipoprotein; ApoA-I, apolipoprotein A-I; SAA, serum amyloid A; OMVs, outer membrane vesicles.

Crucially, the viability of immune cells—particularly tissue-resident macrophages—under such chronic microbial and metabolic stress is strictly dictated by their mitochondrial function [125]. For instance, visceral adipose tissue (VAT)-resident macrophages rely heavily on SerpinB2 to regulate mitochondrial oxidative phosphorylation [125]. This regulation prevents the pathological release of pro-apoptotic cytochrome c from the mitochondria into the cytoplasm by promoting the production of antioxidant glutathione. However, under chronic inflammatory states such as obesity, elevated interferon-γ induces the expression of the transcriptional suppressor Ikaros, which directly binds to the SerpinB2 promoter and downregulates its expression. The ensuing loss of SerpinB2 compromises mitochondrial antioxidant defenses, determining the loss of viability and subsequent decline of this protective macrophage subset. This SerpinB2-regulated mitochondrial injury mechanism provides a core basis for how chronic inflammation dictates macrophage survival, an event closely associated with the development of atherosclerosis. This suggests that bioactive molecules carried by OMVs (including lipids and proteins) may synergize with the loss of protective pathways like SerpinB2 to induce cytotoxicity and inflammatory responses in cardiac and vascular tissues by disrupting mitochondrial homeostasis [126] (Figure 5).

In summary, sphingolipids such as ceramides produced by the gut microbiota (particularly Bacteroides) enter the bloodstream via OMVs as nanoscale transport vehicles and colonise distant organs including the heart [115,127]. This process not only remodels the host’s lipid metabolism profile [116], but may also constitute a significant potential mechanism within the ‘gut–lipid–heart’ axis for inducing cardiovascular pathological damage by targeting mitochondria to induce apoptosis and inflammation [126,128]. To systematically synthesize the complex interactions detailed in the preceding sections, Table 1 provides a comprehensive summary of the key lipid-derived metabolites generated by the gut microbiota. For easier reference tracking, this table catalogs the specific host receptors targeted by each metabolite, their subsequent physiological or pathological effects on lipid metabolism, and their ultimate cardiovascular outcomes, alongside the graded evidence from primary literature.

## 4. Disease Atlas: The Comprehensive Manifestation of the Gut–Adipose Axis in the Cardiovascular Disease Spectrum

### 4.1. Hyperlipidaemia: Initiation Phase

Hyperlipidaemia is a core initiating factor in atherosclerotic cardiovascular disease (ASCVD), with the gut microbiota playing a pivotal role as a “metabolic switch”. Research indicates that dysbiosis of the gut microbiota not only directly causes dyslipidaemia by disrupting cholesterol and triglyceride metabolic pathways, but also exacerbates lipid metabolism disorders by compromising the intestinal barrier function and inducing systemic inflammation.

Delayed postprandial TG clearance, known as postprandial hyperlipidaemia, has been established as an independent risk factor for cardiovascular disease. Specific members of the gut microbiota and their metabolites play crucial roles in regulating intestinal lipid absorption and chylomicron secretion. For instance, certain bacteria (such as *Bacteroides eggerthii*) can downregulate the expression of lipid transporters (e.g., CD36) in intestinal epithelial cells by producing specific metabolites (e.g., 2-hydroxyisocaproic acid, HICA), thereby inhibiting lipid absorption and chylomicron assembly [129]. Conversely, dysbiosis may lead to chylomicron retention or excessive secretion within the intestine, subsequently triggering postprandial hypertriglyceridaemia.

The gut microbiota directly influences serum cholesterol levels by regulating bile acid metabolism and cholesterol excretion. Specific probiotics (such as *Lactobacillus reuteri*) can increase faecal SCFA content, activating butyrate and vitamin B6 metabolic pathways, thereby reducing plasma total cholesterol and low-density lipoprotein cholesterol (LDL-C) levels [130]. Furthermore, certain plant polysaccharides (e.g., *Polygonatum polysaccharides*, *Monascus polysaccharides*) promote the growth of beneficial bacteria (e.g., *Akkermansia*, *Bifidobacterium*) by modulating gut microbiota composition. This subsequently accelerates cholesterol conversion to bile acids and faecal excretion by upregulating key hepatic genes such as CYP7A1 and ABCG5/8, thereby effectively ameliorating hyperlipidaemia [131,132].

Multiple studies have elucidated the integrated regulatory mechanisms of the ‘microbiota–metabolite–target organ’ axis in hyperlipidaemia. For instance, supplementation with Limosilactobacillus mucosae restores intestinal glycerophosphocholine (α-GPC) levels and upregulates the tight junction protein Claudin-1, thereby strengthening the intestinal barrier and mitigating periodontitis and hyperlipidaemia induced by lipid metabolism disorders [133]. Another study found that Bacteroides thetaiotaomicron improves hepatic steatosis and hyperlipidaemia by regulating intestinal-hepatic folate and unsaturated fatty acid metabolism [134]. Moreover, complex traditional Chinese medicine formulations—such as Naoxintong Capsules (a multi-herbal preparation enriched with vascular-protective tanshinones and phenolic acids) and Er Miao San (a fundamental two-herb precursor to Simiaowan containing Phellodendri Chinensis Cortex and Atractylodis Rhizoma)—have been demonstrated to enhance the efficacy of lipid-lowering drugs (e.g., atorvastatin) or directly ameliorate metabolic syndrome-associated hyperlipidaemia by reshaping gut microbiota composition and modulating plasma metabolite profiles (e.g., ether lipid metabolism, propionic acid metabolism) [129,135]. These findings underscore the substantial potential of gut microbiota-targeting interventions in the prevention and management of hyperlipidaemia.

### 4.2. Atherosclerosis: Lipid Deposition in the Vascular Wall

Atherosclerosis is a chronic inflammatory vascular disease whose core pathological processes involve lipid deposition within the vascular wall, inflammatory responses, and plaque instability. Increasing evidence indicates that the oral and gut microbiota play a pivotal role in this process, forming the “oral–gut–vascular axis”.

Research has revealed that periodontal pathogens such as Porphyromonas gingivalis (Pg) can directly invade the vascular wall via the oral-blood route and colonise atherosclerotic plaques. The presence of Pg not only correlates positively with increased necrotic core area within plaques but also significantly exacerbates plaque instability by inducing oxidative stress and necroptosis in macrophages [136]. Mechanistically, Pg and its virulence factors (such as lipopolysaccharide LPS and gingipain) activate the TLR4 signalling pathway within macrophages, leading to FOXO3 protein degradation. This subsequently releases transcriptional repression of the scavenger receptor MSR1, promoting macrophage lipid uptake and ultimately inducing cell death [136]. Furthermore, Pg induces smooth muscle cell apoptosis and inhibits macrophage efferocytosis, leading to the accumulation of apoptotic cells within plaques and further exacerbating the inflammatory microenvironment [137]. The regulatory threshold for this efferocytic capacity is strictly governed by epigenetic modifications. Recent studies reveal that the transcription factor RBPJ acts as a critical epigenetic driver of macrophage efferocytosis by dampening the repressive heterochromatin mark H3K9me3 [138]. This RBPJ-mediated epigenetic remodeling specifically de-represses downstream effectors such as Stard13 and Arsg [138]. The subsequent upregulation of Stard13 triggers a subcellular cascade that amplifies actin polymerization—via the concomitant inhibition of Rho and activation of RAC GTPases—ultimately providing the mechanical force necessary for the engulfment of apoptotic debris [138]. Therefore, the impairment of these highly coordinated epigenetic and cytoskeletal networks by pathogen-derived signals likely constitutes a core mechanism driving efferocytotic failure and plaque vulnerability. Notably, beyond direct infection, Pg accelerates atherosclerosis progression by altering methylation patterns of circadian genes (e.g., BMAL1), thereby intensifying oxidative stress and inflammatory responses in vascular endothelial cells [139].

Beyond *P. gingivalis*, other members of the oral and gut microbiota are also closely associated with the progression of atherosclerosis. For instance, Fusobacterium nucleatum has been found to be enriched within atherosclerotic plaques and can promote inflammation and lipid deposition by activating the PI3K-AKT/MAPK/NF-κB signalling pathways in macrophages [140]. In HIV-infected women, Fusobacterium nucleatum abundance positively correlates with carotid plaque presence and is accompanied by elevated serum inflammatory markers such as CXCL9 [141]. Conversely, the abundance of certain butyrate-producing bacteria (e.g., the genus Roseburia) negatively correlates with plaque burden, suggesting a potential protective role [141]. Recent studies further reveal that increased abundance of the Collinsella genus correlates with fatty liver disease and atherosclerosis, while supplementation with omega-3 fatty acids significantly reduces its abundance, potentially contributing to improved cardiovascular health [55]. In multi-ethnic cohort studies, heightened abundance of Streptococcus species (e.g., *S. anginosus* and *S. oralis*) in the gut showed significant associations with subclinical coronary atherosclerosis and systemic inflammatory markers (e.g., hs-CRP and neutrophil counts), further supporting the hypothesis of oral microbiota colonising the gut and influencing cardiovascular health [142].

In summary, atherosclerosis is no longer regarded as a mere lipid metabolism disorder, but rather as a complex ecosystem dysregulation process involving microbe–host interactions. The direct colonisation by oral pathogens, systemic inflammation triggered by gut microbiota dysbiosis, and the accumulation of specific metabolites (such as TMAO) collectively form a multidimensional network that drives lipid deposition within the vascular wall and promotes plaque instability.

### 4.3. Hypertension: Lipid–Vascular Tension Coupling

Gut microbiota dysbiosis has emerged as a pivotal component in the pathophysiology of hypertension, exerting precise regulation over blood pressure levels via the ‘microbiota–metabolite–vascular axis’. Research indicates that SCFAs, metabolised by gut microbiota, serve as the core messenger linking dietary fibre intake to blood pressure regulation. SCFAs exert hypotensive and cardiovascular protective effects by activating host G protein-coupled receptors (GPCRs) such as GPR41, GPR43, and GPR109A, alongside olfactory receptors Olfr78 and Olfr558 [143,144]. Notably, the Olfr78 receptor is expressed in central cardiovascular regulatory regions such as the paraventricular nucleus (PVN) of the hypothalamus and the rostral ventrolateral medulla (RVLM), mediating the central regulation of blood pressure by SCFAs [145,146]. For instance, gut microbiota-derived acetate can reduce arterial blood pressure by activating the Olfr59 receptor (a homologue of Olfr78) on RVLM neurons, thereby inhibiting sympathetic nerve efferent impulses [146]. Peripherally, although Olfr78 may mediate renin release in renal afferent arterioles to elevate blood pressure, overall SCFA-induced vasodilation via GPR41/43 predominates, ultimately resulting in hypotension [144].

Beyond the direct vascular effects of SCFAs, vascular endothelial dysfunction triggered by lipid metabolism disorders also constitutes a significant driver of hypertension. Ox-LDL plays the role of an “endothelial injurer” in this process. Dyslipidaemia and oxidative stress induced by high-fat diets or dysbiosis (such as enrichment of TMAO-producing bacteria) promote the oxidative modification of LDL into ox-LDL [92]. Ox-LDL induces ROS production by upregulating scavenger receptors on endothelial cells (e.g., CD36 and LOX-1), thereby quenching endothelial nitric oxide (NO) and impairing vasodilatory function and increasing vascular stiffness [143,147]. Research indicates that dietary interventions rich in flavonoids (e.g., pomegranate juice, coriander) or probiotics (e.g., *Limosilactobacillus fermentum*) can reshape the gut microbiota, thereby reducing ox-LDL levels and restoring NO bioavailability. This improves endothelium-dependent vasodilation and effectively alleviates hypertension [147,148]. Furthermore, the gut microbiota-derived isoflavone metabolite 8-pentenylnaringenin (8-PN) has been demonstrated to restore NO production by mediating eNOS phosphorylation via G protein-coupled estrogen receptor (GPER), thereby counteracting angiotensin II-induced endothelial dysfunction [149]. In summary, the complex microbiome-mediated mechanisms underlying hypertension involve both blood pressure regulation through receptors such as Olfr78 and the coupled effects of lipid metabolites (particularly ox-LDL) on vascular tone.

### 4.4. Myocardial Infarction: Thrombosis, Inflammatory Storm, and Lipogenic Reprogramming

Myocardial infarction (MI) is not merely an ischaemic event, but rather a complex systemic pathological process involving the gut microbiota and its metabolic products. Among these, the gut microbe-derived metabolite phenylacetylglutamine (PAGln) has been demonstrated to be a key driver of thrombosis and major adverse cardiovascular events (MACE). PAGln is produced when dietary phenylalanine is broken down by microorganisms (such as Proteobacteria containing the porA gene) into phenylacetic acid (PAA), which is then metabolised by the host liver. Research indicates that PAGln significantly enhances platelet reactivity and aggregation capacity by activating G protein-coupled receptors (GPCRs) on platelets, particularly α2A, α2B, and β2-adrenergic receptors, thereby promoting thrombosis [150,151]. In high-risk coronary heart disease patients, elevated plasma PAGln levels correlate not only with increased thrombotic risk but also serve as an independent predictor of mortality and severe disability following MI [152]. Furthermore, PAGln exacerbates atrial fibrosis and electrical remodelling post-MI by inducing ferroptosis and activating NLRP3 inflammasomes, thereby increasing susceptibility to atrial fibrillation [153].

Following MI, haemodynamic alterations induced by cardiac injury lead to intestinal hypoperfusion and compromised barrier function, thereby opening Pandora’s box for gut microbiota translocation. Research indicates that post-MI increased intestinal permeability permits bacterial entry (particularly from the phylum Proteobacteria) and their products (such as LPS) into the bloodstream, triggering systemic inflammatory storms that in turn exacerbate myocardial ischaemia–reperfusion (I/R) injury [154,155]. Eradicating the gut microbiota via antibiotic cocktail therapy or enhancing intestinal barrier function with GLP-2 can block bacterial translocation, thereby reducing systemic inflammation and myeloid cell mobilisation. This significantly improves infarct size and cardiac function following myocardial infarction [154]. Moreover, beneficial microbiota metabolites such as indole-3-propionic acid (IPA), whose levels decline post-MI, can be supplemented to inhibit ferroptosis in cardiomyocytes via the NRF2/GPX4 axis, thereby protecting the heart from I/R injury [156]. Conversely, oral–intestinal translocation of certain pathogenic bacteria (e.g., *Fusobacterium nucleatum*) exacerbates post-MI myocardial injury by promoting B-cell production of pro-inflammatory factors such as IL-6 and TNF-α [157].

Crucially, this post-MI inflammatory storm is inextricably linked to localized metabolic reprogramming within immune cells, establishing a deleterious “lipid–macrophage–fibrosis” axis that drives adverse cardiac remodeling. While systemic microbial translocation and metabolites initiate the inflammatory cascade, the sustained structural deterioration is heavily regulated by the intracellular lipid metabolism of resident and infiltrating macrophages. Recent spatial multi-omics and single-cell RNA sequencing studies reveal that cardiac macrophages residing in the infarcted myocardium undergo profound lipogenic reprogramming, characterized by the activation of de novo fatty acid synthesis and the marked upregulation of key metabolic enzymes, including ATP citrate lyase (ACLY) and fatty acid synthase (FASN). This enhanced fatty acid synthesis acts as a potent driver of pathological remodeling rather than a mere metabolic byproduct. Mechanistically, ACLY acetylates the promoter region of the upstream regulator Krt17, which subsequently drives the robust production of pro-fibrotic cytokines, notably IL-33. This macrophage-derived signaling directly orchestrates the specific expansion of a highly fibrogenic fibroblast subpopulation (Fibroblast 5), defined by high extracellular matrix gene expression. Consequently, the targeted silencing or myeloid-specific ablation of Acly and Fasn effectively confines this pathogenic fibroblast expansion, significantly reducing adverse post-infarction fibrosis and improving overall cardiac function [158]. This paradigm highlights that beyond systemic lipid toxicity, the targeted inhibition of macrophage fatty acid synthesis pathways represents a critical intervention node to disrupt the cross-talk between lipid metabolism, inflammation, and irreversible cardiac remodeling.

### 4.5. Heart Failure: Lipotoxicity and Energy Metabolism

Within the complex pathophysiological landscape of heart failure, energy metabolism remodelling and lipid toxicity represent two core driving factors, with the gut microbiota and its metabolites playing a pivotal regulatory role. The hearts of heart failure patients frequently endure a state of ‘energy starvation’, characterised by diminished mitochondrial oxidative capacity in the myocardium, resulting in inadequate energy supply [159]. Against this backdrop, gut microbiota dysbiosis may exacerbate the myocardial energy crisis. For instance, the gut-derived metabolite N, N, N-trimethyl-5-aminovaleric acid (TMAVA) has been shown to inhibit γ-butyroxybutyric acid hydroxylase (BBOX), thereby blocking endogenous carnitine synthesis. This subsequently reduces long-chain fatty acid oxidation, leading to myocardial lipid accumulation and hypertrophy [160]. This TMAVA-induced impairment of fatty acid oxidation and lipid toxicity constitutes a key mechanism in the progression of cardiac hypertrophy and heart failure.

**Figure 6:**
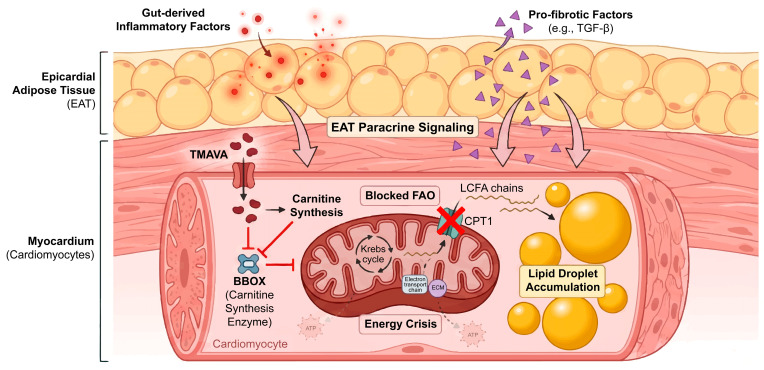
Disease-Specific Map—Energy Metabolic Collapse & Ectopic Fat in Heart Failure. EAT, epicardial adipose tissue; TGF-β, transforming growth factor β; TMAVA, N,N,N-trimethyl-5-aminovaleric acid; BBOX, γ-butyrobetaine hydroxylase; CPT1, carnitine palmitoyltransferase 1; LCFAs, long-chain fatty acids; ATP, adenosine triphosphate; FAO, fatty acid oxidation.

Heart failure with preserved ejection fraction (HFpEF) is particularly prevalent in populations with metabolic syndrome and obesity, characterised by systemic inflammation and metabolic dysfunction. Research indicates that in HFpEF patients and animal models, gut microbiota dysbiosis is closely associated with alterations in plasma metabolites such as trimethylamine-N-oxide (TMAO) and SCFA [161]. Notably, in obesity-associated HFpEF, the epicardial adipose tissue (EAT)—functioning as an active metabolic organ—may secrete pro-inflammatory and pro-fibrotic factors in response to gut metabolites, directly impacting adjacent myocardial tissue [162]. Furthermore, a high-choline diet, converted to TMAO via the gut microbiota, exacerbates left ventricular hypertrophy, pulmonary congestion, and diastolic dysfunction in HFpEF models. Inhibiting TMA production significantly alleviates these cardiac remodelling processes [163] (Figure 6).

Intervention strategies targeting these metabolic abnormalities demonstrate therapeutic potential. For instance, supplementing with specific probiotics (such as *Lactobacillus*) or their metabolites (such as 12-HEPE) can modulate the gut microbiota, thereby mitigating inflammatory responses and fibrosis following myocardial infarction [164]. Similarly, improving systemic and myocardial energy metabolism through pharmacological interventions (e.g., SGLT2 inhibitors) or dietary modifications (e.g., ketogenic diet, essential amino acid supplementation) has been demonstrated to effectively alleviate heart failure symptoms [165,166]. These findings underscore the importance of the gut–heart axis in regulating myocardial energy metabolism and lipid toxicity, and provide novel metabolic targets for the prevention and treatment of heart failure.

### 4.6. Abdominal Aortic Aneurysm: Lipid-Driven Matrix Degradation

The formation and progression of abdominal aortic aneurysms (AAAs) represent a complex pathological process involving vascular wall structural remodelling, inflammation, and oxidative stress, within which the gut microbiota and its metabolites play a pivotal regulatory role. Research indicates that dysbiosis of the gut microbiota drives vascular wall disruption characterised by elastin degradation by modulating lipid metabolism and matrix metalloproteinase (MMP) activity. Notably, TMA-producing gut bacteria, such as *Clostridium* and *Lachnoclostridium*, are strongly associated with AAA risk. Elevated levels of their metabolite, TMAO, have been demonstrated to be an independent predictor of AAA occurrence and rapid expansion [167]. TMAO induces the unfolded protein response (UPR) by upregulating the endoplasmic reticulum stress kinase PERK in vascular smooth muscle cells, thereby promoting inflammation and apoptosis, which compromises vascular wall integrity [167].

Matrix metalloproteinases (MMPs), particularly MMP-2 and MMP-9, are key effector molecules responsible for the rupture of elastic fibres in the aortic wall’s media and the formation of AAA. The gut microbiota can modulate MMP activity through multiple pathways. On the one hand, certain commensal bacteria (such as *Hungatella hathewayi*) can protect vascular walls from damage by producing taurine, thereby inhibiting inflammation and MMP-mediated degradation of vascular smooth muscle cells [168]. Conversely, dysbiotic metabolic aberrations, such as indole sulphate (IS) and phenylacetylglutamine (PAGln), may upregulate MMP expression and enhance their activity by activating the AhR or adrenergic receptor signalling pathways, thereby accelerating matrix degradation [169]. Furthermore, reduced abundance of butyrate-producing bacteria (e.g., *Roseburia intestinalis*) correlates with increased formation of neutrophil extracellular traps (NETs) in AAA patients, wherein NET-released proteases (e.g., MMP-9) constitute key drivers of matrix destruction [170].

Oxidised lipids play an “ignition” role in the pathophysiology of AAA, particularly in activating inflammation within the vascular adventitia. The accumulation of oxidised low-density lipoprotein (ox-LDL) and other lipid peroxidation products, such as 4-HNE, within aneurysmal tissue activates fibroblast and immune cells (including macrophages and T cells) in the vascular adventitia, triggering a robust inflammatory response [167,170]. This inflammatory environment not only promotes the secretion and activation of matrix metalloproteinases (MMPs) but also induces phenotypic transformation and apoptosis in vascular smooth muscle cells, further compromising the structural integrity of the arterial wall. Research indicates that specific gut microbiota (e.g., *Bifidobacterium*) and their metabolites (e.g., 12-HEPE) can mitigate this lipid-driven adventitial inflammation by regulating lipid metabolism and anti-inflammatory signalling pathways, thereby slowing AAA progression [133].

Beyond remote regulation via metabolites, direct colonisation of bacterial DNA within the aneurysm wall is also considered a potential mechanism for AAA pathogenesis. Although evidence for the presence of viable bacteria within AAA tissue remains inconclusive, multiple studies have detected DNA sequences of periodontal pathogens (such as *Porphyromonas gingivalis*) and other bacteria (such as *Burkholderia*) in aneurysm wall samples [171,172]. These bacterial components may act as pathogen-associated molecular patterns (PAMPs), activating local innate immune responses via TLRs. This induces the expression of inflammatory cytokines and MMPs, thereby promoting lipid oxidation and foam cell rupture, accelerating plaque instability and aneurysm expansion [167,170]. This ‘Trojan horse’ mechanism of bacterial DNA colonisation offers a novel perspective for understanding the infectious aetiology of AAA.

### 4.7. Atrial Fibrillation: The Proarrhythmic Effect of Epicardial Fat

The pathogenesis of atrial fibrillation (AF) extends beyond the cardiac electrophysiological remodelling itself, being closely associated with gut microbiota dysbiosis and the inflammatory state of EAT, forming a pathological ‘gut–fat–atrial fibrillation’ axis. Research indicates that gut dysbiosis and the abnormal metabolism of its byproducts constitute significant triggers for AF [173,174]. In AF patients, reduced abundance of beneficial gut bacteria (such as short-chain fatty acid-producing flora) coincides with increased prevalence of pathogenic bacteria (e.g., LPS-producing Gram-negative bacteria), leading to compromised intestinal barrier function and metabolic endotoxinaemia [173,175]. Elevated circulating LPS and glucose levels activate NLRP3 inflammasomes in atrial tissue and EAT, subsequently triggering fibrosis and electrophysiological abnormalities that promote AF onset [176]. Crucially, the clinical assessment of this fibrotic remodeling heavily relies on precise left atrial fibrosis quantification, typically achieved through late gadolinium enhancement magnetic resonance imaging (LGE-MRI) or high-density electroanatomical voltage mapping. These quantitative imaging and mapping modalities have revealed that the extent of left atrial fibrotic burden correlates directly with regional EAT volume and its localized inflammatory activity, serving as a critical structural substrate for AF initiation and maintenance [177,178,179]. Notably, as a metabolically active tissue adjacent to the myocardium, the inflammatory state of EAT exerts direct paracrine effects on atrial electrophysiology. In rapid atrial pacing-induced AF models, EAT exhibits marked inflammatory responses (e.g., elevated IL-6 and TNF-α levels) and adipokine dysregulation (e.g., reduced adiponectin levels), which further exacerbate atrial fibrosis and arrhythmia susceptibility [180].

Furthermore, the causality within the EAT-AF axis is fundamentally bidirectional, establishing a vicious pathogenic cycle. While pathological EAT initially promotes AF via inflammatory paracrine signaling and fatty acid infiltration, the onset of AF itself drives reciprocal deleterious changes within EAT. The rapid atrial rates, altered hemodynamics, and mechanical stretch inherent to AF induce the myocardial release of profibrotic and proinflammatory cytokines, which subsequently promote EAT expansion, local lipid dysregulation, and a phenotypic shift toward a hyper-inflammatory state. This bidirectional crosstalk permanently alters the local microenvironment, accelerating structural and electrophysiological deterioration, firmly supporting the “AF begets EAT” theory [180,181].

Moreover, gut microbiota metabolites such as TMAO and SCFAs play dual roles in the pathogenesis of AF. Elevated TMAO levels correlate closely with AF progression, potentially through exacerbating cardiac inflammation and connexin remodelling [182]. Conversely, SCFAs (such as acetate, propionate, and butyrate) exhibit protective effects. SCFAs can reverse atrial structural and electrical remodelling by activating G protein-coupled receptors (e.g., GPR43), inhibiting NLRP3 inflammasome and HDAC activity, thereby reducing inflammatory responses, alleviating endoplasmic reticulum stress, and improving mitochondrial function [173,183]. In sepsis-induced AF models, pharmacological interventions (e.g., GTS-21 or metformin) successfully suppressed inflammation-mediated atrial remodelling and AF susceptibility by modulating macrophage polarisation or enhancing adipokine signalling (e.g., adiponectin) in EAT [180,184]. These findings underscore the role of EAT as a relay station in gut-to-heart signalling pathways and suggest that modulating the ‘gut–adipose–atrial fibrillation’ axis may offer novel strategies for the prevention and treatment of AF. We also conduct a comparative analysis of the differences in microbiome mechanisms across various diseases. To synthesize the diverse pathological manifestations discussed across this disease atlas, Table 2 delineates the specific microbial signatures and core lipid pathologies associated with each cardiovascular condition. This table serves as a comparative reference guide, explicitly mapping the foundational studies to their respective disease states to track how distinct microbiome alterations drive disease-specific mechanisms.

## 5. Intervention Strategy: Lipid Management Targeting the Microbiome

### 5.1. Next-Generation Probiotics

With the deepening understanding of the gut–liver axis mechanism, the development of next-generation probiotics (NGPs) has expanded beyond traditional lactic acid bacteria to include symbiotic bacteria with specific metabolic functions. These aim to manage metabolic syndrome by precisely regulating lipid metabolic pathways.

BSH activity serves as a key indicator for screening lipid-lowering probiotics. Strains exhibiting high BSH activity can release glycine or taurine conjugates from conjugated bile acids, thereby increasing the proportion of unconjugated bile acids. This promotes bile acid excretion and modulates the FXR signalling pathway. Research indicates that Lactobacillus reuteri ZJ617 not only exhibits high BSH activity but also serves as a substrate source to stimulate intestinal microbiota synthesis of spermidine. This synergistic effect not only restores gut microbiota diversity but also effectively mitigates high-fat diet-induced obesity, hyperlipidaemia, and insulin resistance. The mechanism involves spermidine’s direct regulation of host metabolism [185]. Furthermore, other probiotics with high BSH activity, such as *Lactiplantibacillus plantarum* KABP011-013, have demonstrated significant cholesterol-lowering effects in clinical trials. By increasing faecal bile acid excretion and reducing plasma FGF19 levels, they improved the lipid profile in overweight subjects [186].

*Akkermansia muciniphila* (AKK bacteria), as a mucin-degrading bacterium, has been widely recognised as a “star” NGP for improving metabolic health. It not only enhances intestinal barrier function and reduces metabolic endotoxinaemia but also optimises blood lipid profiles through multiple mechanisms. Firstly, AKK bacteria or its pasteurised form (Pasteurised AKK) and its outer membrane protein Amuc_1100 significantly increase energy expenditure and reduce fat accumulation without altering food intake [187]. Secondly, AKK effectively reverses hepatic steatosis and inflammation associated with metabolic dysfunction-associated fatty liver disease (MAFLD) by regulating the gut–liver axis, particularly through promoting hepatic mitochondrial oxidation and L-aspartate metabolism [188]. More importantly, AKK bacteria can also activate the SIRT1 signalling pathway by modulating gut microbiota-derived metabolites (such as 4-hydroxyphenylacetic acid and short-chain fatty acids), thereby promoting the beige-tissue conversion and thermogenesis of white adipose tissue, leading to systemic improvements in obesity and insulin resistance [189]. The exceptional performance of AKK in restoring intestinal barrier integrity, reducing serum LPS levels, and inhibiting the TLR4/NF-κB inflammatory pathway positions it as a promising strain for preventing and treating cardiovascular diseases and metabolic syndrome [190,191].

Represented by strains with high BSH activity and AKK bacteria, the new generation of probiotics demonstrates significant potential in lipid management and the treatment of metabolic disorders. This is achieved through direct involvement in bile acid metabolism, strengthening the intestinal barrier, regulating host energy expenditure, and modulating anti-inflammatory signalling pathways.

### 5.2. Molecularly Targeted Drugs

Developing molecularly targeted drugs that act upon key enzymes and signalling pathways within the gut microbiota–host metabolic axis has emerged as a novel strategy for lipid management. Among these approaches, strategies inhibiting TMAO production have garnered significant attention. TMA lyase serves as the pivotal enzyme by which gut bacteria convert dietary precursors such as choline and carnitine into TMA. Novel covalent inhibitors, such as derivatives of DMB (3,3-dimethylbutanol) or fluoromethylcholine (FMC), can specifically target the active site of TMA lyase. These agents significantly reduce TMA and TMAO levels in vivo without ostensibly affecting microbial growth, thereby inhibiting atherosclerotic thrombosis in preclinical models [192,193].

However, despite these promising preclinical outcomes, the clinical translation of TMA lyase inhibitors has largely failed to materialize to date [194]. A primary obstacle to this translation is the risk of significant off-target effects. For example, the targeted blockade of TMA lyase leads to the intestinal accumulation of un-metabolized precursors, such as choline and L-carnitine, which may paradoxically activate alternative, potentially detrimental host metabolic cascades or be metabolized by non-targeted microbial pathways [195]. Additionally, the potential for systemic absorption of these inhibitors raises concerns regarding inadvertent interference with structurally homologous mammalian enzymes [196].

Furthermore, the long-term gut ecological consequences of chronic TMA lyase inhibition remain a critical and under-evaluated concern [197]. Although these inhibitors are designed as non-lethal agents, blocking a major nutrient utilization pathway fundamentally alters the ecological fitness of TMA-producing microbial taxa. Over time, this targeted metabolic deprivation exerts an artificial selective pressure that could disrupt broader microbiome homeostasis. Such interventions risk triggering compensatory shifts in the microbial architecture, potentially driving the overgrowth of alternative opportunistic pathogens or inadvertently diminishing the abundance of beneficial commensals that rely on shared cross-feeding networks [198].

To bypass these upstream challenges, inhibitors targeting downstream signalling pathways exhibit therapeutic potential against TMAO-induced inflammation and vascular injury. For instance, TMAO exacerbates atrial remodelling and arrhythmias by activating the PERK/IRE1α/NLRP3 axis, whereas sodium butyrate effectively suppresses this endoplasmic reticulum stress pathway, exerting cardioprotective effects [199].

Traditional bile acid sequestrants (such as cholestyramine and colesevelam) have been found to possess ‘atypical’ effects beyond lowering serum cholesterol by inhibiting bile acid reabsorption, including reshaping the gut microbiota and improving metabolic health. Research indicates that co-administration of colesevelam, whilst promoting cholesterol clearance through brown fat activation (e.g., via β3-adrenergic receptor agonists), prevents excessive plasma bile acid accumulation. This further lowers serum total and non-HDL cholesterol levels and significantly reduces atherosclerotic plaque area [200]. This combined strategy not only optimises lipid metabolism by promoting cholesterol conversion to bile acids and excretion, but may also exert synergistic anti-inflammatory and metabolic syndrome-improving effects by indirectly modulating gut microbiota composition through altering the composition of the bile acid pool within the intestine. Furthermore, inhibitors targeting bile acid transporters (such as ASBT) are under development, aiming to treat bile acid-related metabolic disorders by regulating the enterohepatic circulation [201].

Beyond TMAO and bile acid pathways, drug development targeting other microbiota-associated metabolic pathways is also advancing. For instance, modulators of the FGF15/19 axis (such as Aldafermin) have demonstrated potential in clinical trials for non-alcoholic steatohepatitis (NASH) to reduce hepatic fat content and improve fibrosis [202]. Concurrently, agonists targeting secondary bile acids produced by gut metabolism (e.g., hydroxycholanic acid) and their receptors (e.g., TGR5, FXR) have been demonstrated to improve fatty liver and metabolic syndrome by modulating lipid oxidation and anti-inflammatory pathways [203,204]. These findings suggest that developing more targeted and effective lipid management therapeutics is achievable by precisely targeting key nodes within the ‘microbiota–metabolite–host’ interaction network.

### 5.3. Diet and Phytochemicals

Diet constitutes the most potent environmental driver for reshaping the gut microbiota to improve lipid metabolism. The Mediterranean diet, recognised as a healthy dietary pattern, derives its cardiovascular benefits largely from gut microbiota regulation. Studies indicate that Mediterranean dietary interventions significantly reduce plasma cholesterol and faecal bile acid levels, increase fibre-degrading bacteria (such as *Faecalibacterium prausnitzii*) abundance, and promote beneficial metabolites like urinary stercospermine [205]. Notably, extra virgin olive oil intake correlates positively with gut microbiota diversity, potentially enhancing cognitive function and metabolic health by enriching specific beneficial genera such as Adlercreutzia [206]. Moreover, polyphenols widely present in plant-based foods (e.g., resveratrol, tea polyphenols), despite their low bioavailability, exert significant ‘prebiotic-like effects’ in the colon. For instance, resveratrol undergoes gut microbiota metabolism to produce 4-hydroxyphenylacetic acid (4-HPA), a metabolite that activates the SIRT1 signalling pathway. This induces browning and thermogenesis in white adipose tissue, thereby systemically improving obesity and dyslipidaemia [189]. Phytosterols, meanwhile, can reduce plasma endothelin-1 levels and improve vascular endothelial function by modulating the microbiota, independently of their LDL-C-lowering effects [207].

Beyond dietary polyphenols, numerous phytochemicals derived from traditional Chinese medicine (TCM) demonstrate more specific potential for modulating the microbiota. Given the vast array of plant-derived compounds with microbiome-modulating properties, Table 3 systematically catalogs these natural phytochemicals to facilitate easier tracking of the supporting literature. It highlights their primary botanical sources, specific microbial targets, and subsequent host metabolic benefits. For instance, alkaloid compounds such as berberine have been demonstrated to specifically inhibit the growth of trimethylamine-producing bacteria and CutC enzyme activity, thereby blocking the generation of prothrombotic metabolites like TMAO and PAGln at their source, exerting anti-atherosclerotic effects [208,209]. Nuciferine, meanwhile, repairs the intestinal barrier by reducing the abundance of LPS-producing Desulfovibrio species, thereby blocking metabolic endotoxemia [210]. Tilianin from Schisandra chinensis modulates the gut microbiota to upregulate the hepatic SREBP2-LDLR pathway [211]. Hyperoside, meanwhile, inhibits bacterial BSH activity to increase conjugated bile acids, thereby activating the FXR-FGF15 axis [212]. Moreover, integrated traditional Chinese medicine formulations (such as the aforementioned Naoxintong and Er Miao San) demonstrate overall efficacy in improving metabolic syndrome by multi-targeted remodelling of the gut microbiota structure and precisely regulating short-chain fatty acid and bile acid metabolic pathways [132,135].

### 5.4. The Microbial Mechanisms of Bariatric Surgery

Weight-loss surgery, particularly Roux-en-Y gastric bypass (RYGB) and sleeve gastrectomy (SG), is considered the most effective treatment for severe obesity and its associated metabolic complications, such as type 2 diabetes and hyperlipidaemia. Beyond substantial weight reduction, these procedures deliver rapid and sustained improvements in metabolic health, with profound reshaping of the gut microbiota and its metabolic products playing an indispensable role in this process [213,214,215]. Surgical alterations to anatomical structures—such as small intestinal bypass, changes in acidity, and variations in oxygen content—create novel ecological niches for gut microbes, triggering substantial shifts in microbial composition and function [216,217].

Post-RYGB, alterations in gut microbiota correlate closely with increased GLP-1 (glucagon-like peptide-1) secretion, considered a key mechanism for rapid post-operative normalisation of blood glucose and lipids. Studies indicate heightened abundance of Veillonella, Streptococcus and other bacteria in the gut post-RYGB, with these changes positively correlated to elevated GLP-1 levels [218]. Microbiota transplantation experiments confirm that post-operative microbiota can independently improve host glucose and lipid metabolism by enhancing GLP-1 secretion from intestinal L cells, irrespective of weight loss [214]. GLP-1 not only promotes insulin secretion but also suppresses appetite, delays gastric emptying, and directly influences lipid metabolic pathways to facilitate postprandial triglyceride clearance [219,220]. Furthermore, novel endoscopic devices (e.g., ForePass) mimicking biliopancreatic diversion effects similarly demonstrated elevated GLP-1 levels and metabolic improvements, accompanied by increased *Akkermansia muciniphila* abundance. This further underscores the central role of the microbiota–gut hormone axis in the metabolic benefits of surgical interventions [221].

**Figure 7:**
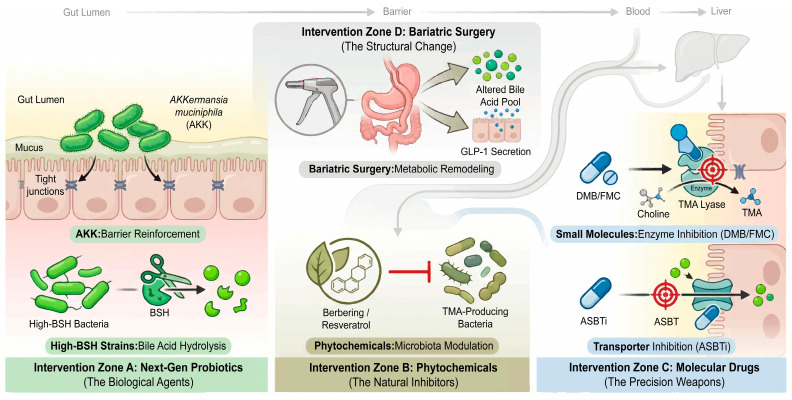
Therapeutic Strategy Overview—Multidimensional Targets for Microbiome Intervention. AKK, *Akkermansia muciniphila*; BSH, bile salt hydrolase; TMA, trimethylamine; DMB/FMC, 3,3-dimethyl-1-butanol/fluoromethylcholine; ASBT, apical sodium-dependent bile acid transporter; GLP-1, glucagon-like peptide-1.

Bariatric surgery also reshapes the body’s bile acid profile by altering gut microbiota metabolism of bile acids. Postoperatively, levels of primary bile acids (such as cholic acid and chenodeoxycholic acid) decrease, while secondary bile acids (such as lithocholic acid, deoxycholic acid, and their conjugated forms) increase [216,222,223]. Notably, tauro-deoxycholic acid (TDCA) exhibits a marked increase in concentration within the postoperative small intestine. Acting as an endogenous inhibitor, TDCA suppresses the bacterial bile acid-induced operon, thereby reducing LCA production. This, in turn, improves glucose tolerance by modulating the TGR5 signalling pathway [222]. Another study revealed that post-operative enrichment of Clostridia-related bacteria promotes the generation of conjugated secondary bile acids (e.g., GDCA, TDCA). These bile acids alleviate obesity by activating TGR5 receptors in adipose tissue, thereby enhancing lipolysis and fatty acid oxidation [223]. This microbiota-mediated alteration in the bile acid profile not only influences energy absorption but also acts as a signalling molecule regulating systemic metabolic homeostasis, constituting a crucial molecular basis for metabolic improvements following bariatric surgery [213,222,224] (Figure 7).

## 6. Conclusions & Perspectives

With the emergence of the holobiont theory, the gut microbiota has evolved from a mere “nutritional passenger” to an indispensable “metabolic organ” within the human body. This paper systematically elucidates how the gut microbiota establishes a sophisticated ‘gut–lipid–heart’ regulatory axis through neural, endocrine, and immunometabolic networks. The discovery of this axis not only reshapes our understanding of the pathophysiology of cardiovascular disease (CVD) but also provides a novel strategic vantage point that may help address current therapeutic bottlenecks.

At the physiological homeostasis level, the gut microbiota exhibits sophisticated metabolic regulatory capabilities. BSH derived from the microbiota modifies the bile acid pool, remotely modulating the hepatic FXR-FGF15/19 axis to form a crucial negative feedback loop that helps inhibit endogenous lipid synthesis [23,225]. SCFAs fulfil dual roles as both energy substrates and signalling molecules, finely tuning systemic energy allocation and insulin sensitivity through epigenetic modifications and receptor activation [29,226]. More significantly, the gut microbiota contributes to rapid neuroendocrine regulation of feeding behaviour and postprandial lipid clearance by activating the vagus nerve–hypothalamic reflex arc and promoting the secretion of gut hormones such as GLP-1 [45,58]. However, when microbial dysbiosis occurs, this symbiotic network can transform into a pathogenic driver. The microbial metabolite TMAO has been shown to accelerate atherosclerotic plaque formation and instability by inhibiting macrophage cholesterol reverse transport and inducing endoplasmic reticulum stress [72,84]. LPS translocation resulting from compromised intestinal barrier function not only induces HDL dysfunction but also triggers systemic low-grade inflammation [89]. From the initiation of hyperlipidaemia, through abnormal vascular tension regulation in hypertension, to the thrombus storm following myocardial infarction and the collapse of energy metabolism in heart failure, the pathological imprint of the gut microbiota appears to span much of the lifecycle of cardiovascular disease [129,146].

In the stratified management of cardiovascular risk, gender differences represent a long-underestimated variable. Epidemiological data indicate that premenopausal women exhibit significantly lower CVD risk than men, with the underlying microbiological basis potentially residing in a unique ‘oestrogen–gut microbiota–lipid metabolism’ interactive network. Premenopausal high-oestrogen levels enrich bacteria possessing β-glucuronidase (gmGUS) activity, promoting oestrogen enterohepatic circulation and establishing an oestrogen–microbiota positive feedback loop [227,228]. This positive feedback not only sustains oestrogen’s anti-inflammatory effects but also may help optimize blood lipid profiles by inhibiting intestinal cholesterol absorption and promoting bile acid excretion [229]. However, the postmenopausal ‘masculinisation’ of gut microbiota structure (manifested as increased Firmicutes/Bacteroides ratio) is frequently associated with metabolic phenotype deterioration, serving as a potential early warning sign for steeply rising cardiovascular risk [228]. Furthermore, gender dimorphism manifests in differential metabolism of environmental toxins. Female-specific microbial structures (e.g., enriched Desulfovibrio) enhance absorption of lipophilic pollutants like PCBs, suggesting women may face heightened environmentally related metabolic risks [57]. Consequently, future CVD prevention strategies should consider incorporating a gender perspective, particularly for postmenopausal women. Supplementing with phytoestrogens or specific probiotics (e.g., *Lactobacillus*) to mimic oestrogen’s microbiome effects—thereby enhancing SCFA and oestradiol production to improve osteoporosis and lipid metabolism—represents a highly promising intervention direction [230,231,232].

Faced with vast datasets from the microbiome and host metabolic networks, artificial intelligence (AI) and machine learning (ML) technologies are emerging as new engines for decoding complexity. On one hand, by integrating metagenomics and lipidomics data, novel AI models have demonstrated multimodal risk prediction capabilities that could complement traditional clinical scores such as DASH. For instance, ML models based on faecal metabolomics can predict ASCVD risk with high accuracy (AUC = 0.86) and help distinguish recovery trajectories in acute coronary syndrome patients [233,234]. On the other hand, generative AI is accelerating the development of microbe-targeted therapies and drug design. Models such as ProteoGPT have successfully mined novel antimicrobial peptides from metagenomic data. These molecules show the potential to target drug-resistant pathogens without significantly disrupting overall microbial balance, offering promising therapeutic avenues for CVD patients at risk of infection [235,236].

Despite these promising mechanisms, the clinical translation of microbiome-targeted therapies remains hindered by contradictory data and negative trial outcomes. Interventions exhibiting striking efficacy in murine models—such as specific next-generation probiotics—frequently yield statistically insignificant metabolic improvements in human trials, largely due to colonization resistance and profound inter-individual microbiome variability. Although genetic background is difficult to alter, the high plasticity of the gut microbiota renders it one of the most promising modifiable factors in CVD management. Future clinical translation should focus on multidimensional precision intervention pathways. A key objective could be to transition from traditional ‘probiotic supplementation’ to ‘customised functional strains,’ utilising next-generation probiotics (such as *Akkermansia muciniphila* and strains with high BSH activity) or engineered microbiota to target and repair patient-specific metabolic defects (e.g., elevated TMAO, abnormal bile acid metabolism) [185,188]. Concurrently, clinical practice would benefit from reassessing ‘drug–microbiota’ interactions. The efficacy of medications like statins and antidiabetics may partly stem from their ability to reshape the microbiota, while microbial metabolism also significantly influences drug bioavailability. Future drug development should therefore incorporate ‘microbiota-friendliness’ as a key consideration [57]. Moreover, diet could be increasingly regarded as a ‘therapeutic prescription.’ Building upon the metabolic benefits of Mediterranean and ketogenic diets, medical nutrition therapy (MNT) protocols enriched with specific phytochemicals (e.g., berberine, resveratrol) should be developed to help mitigate lipid metabolism disorders by reshaping microbial communities [206,208]. Ultimately, integrating host genetics with microbiomics to establish a multidimensional omics scoring system, and formally incorporating gut microbiota biomarkers into early CVD screening and prognostic assessment systems, could represent a pivotal step in shifting from a disease-treatment to a disease-prevention strategy [77].

In summary, the elucidation of the gut microbiota–lipid metabolism–cardiovascular health axis not only reveals a multidimensional disease landscape but also furnishes us with a key to unlocking the door to precision cardiovascular medicine. By integrating gender differences, artificial intelligence, and multi-omics approaches to establish a comprehensive microbial ecosystem management system spanning the entire lifespan, we can pave the way for more effective management of cardiovascular disease—the world’s foremost killer.

## Figures and Tables

**Table 1:** Key lipid-derived metabolites from the gut microbiota and their cardiovascular effects.

Target/Receptor	Effect on Lipid Metabolism	CV Outcome	Evidence Level & PMIDs
FXR/TGR5/FGF15/19	Inhibits de novo synthesis of hepatic lipids (SREBP-1c); promotes fatty acid oxidation	Improve hyperlipidaemia; alleviate fatty liver	Level I & II: 39205654 Level II: 41622997, 41146521, 41075893
ACSS2/AMPK/GPR43	Dual function: lipid synthesis substrate vs. AMPK activation to promote oxidation	Bidirectional Regulation of Atherosclerosis (AS) and Non-Alcoholic Fatty Liver Disease (NAFLD)	Level II: 41500198, 40127788, 40041901
GPR41 (FFAR3)	Downregulation of the enterohepatic cholesterol transporter (Npc1l1); inhibition of cholesterol absorption	Lowering total cholesterol and triglycerides	Level II: 41395766, 38889450
HDAC3/GPR43	Epigenetic modifications; Enhancement of mitochondrial fatty acid oxidation (FAO)	Inhibit inflammation; improve insulin resistance	Level I & II: 38810839 Level II: 36372481
PERK/FoxO1/Scavenger Receptors	Inhibits reverse cholesterol transport (RCT); Upregulates CD36 to promote lipid uptake	Promotes foam cell formation; exacerbates atherosclerosis and thrombosis	Level II: 34492674, 38622667, 34753029 Level III: 41264872
AhR	Induces endothelial oxidative stress; promotes VCAM-1 expression	Vascular endothelial dysfunction; accelerated vascular sclerosis	Level II: 35202128 Level III: 32260489
Adrenergic receptors (α_2_A, α_2_B, β_2_)	Enhanced platelet reactivity and aggregation	Promotes thrombosis; increases the risk of myocardial infarction (MI)	Level I: 39466896 Level III: 40217126, 32210404
TLR4/inflammasome	Induction of SAA replacement for ApoA-I in HDL (dysfunctional HDL)	Systemic inflammation; Atherosclerotic plaque instability	Level II: 32970631 Level III: 40894318

Evidence grading is categorized as follows: Level I indicates primary clinical studies (e.g., randomized controlled trials, prospective cohorts, or analyses of human patient samples); Level II indicates primary preclinical/mechanistic studies (e.g., in vivo animal models, fecal microbiota transplantation, and in vitro molecular mechanisms); Level III indicates review articles, perspectives, and non-mechanistic associative literature. PMIDs classified as ‘Level I & II’ encompass translational studies validating mechanisms in both human cohorts and animal models.

**Table 2:** Pathological Specificity of the Gut–Fat–Heart Axis in Different Cardiovascular Diseases.

CVD Type	Microbial Signature	Core Lipid Pathology	Key Mechanism	Evidence Level & PMIDs
Hyperlipidemia	Bacteroides eggerii (HICA producer); Lactobacillus reuteri	Delayed postprandial TG clearance; Impaired cholesterol excretion	Downregulation of CD36 inhibits absorption; Modulation of CYP7A1/ABCG5 regulates transformation and excretion.	Level I: 41544465 Level II: 41556761, 35334930
Atherosclerosis	Porphyromonas gingivalis (Pg); Fusobacterium	Intimal lipid deposition; Macrophage foaming	Oral bacterial ectopic colonisation; TLR4 activation induces necroptosis	Level I & II: 40404630 Level II: 37100345, 32078488
Hypertension	Reduction in SCFA-producing bacteria; Increase in TMA-producing bacteria	Oxidised LDL (ox-LDL) damages the endothelium; Abnormal regulation of vascular tone	Olfr78/GPR41 signal imbalance; ox-LDL quenches NO	Level II: 40345351, 41254951 Level III: 35932976
Myocardial Infarction	Proteobacteria phylum amplification (post-MI); Fusobacterium	Platelet hyperreactivity; Ischemia–reperfusion injury	PAGin activates adrenergic receptors; Intestinal barrier disruption triggers an inflammatory storm.	Level I: 39466896 Level I & II: 36715640 Level III: 40217126
HFpEF	TMAVA-producing bacteria enrichment	Impaired myocardial fatty acid oxidation; accumulation of lipid toxins	TMAVA inhibits BBOX enzyme, blocking carnitine synthesis; mitochondrial dysfunction	Level I & II: 35365608 Level II: 32417378
AAA	Clostridium; P. gingivalis	Degradation of elastin in the vascular wall; Lipid-driven matrix destruction	The TMAO/AhR axis upregulates MMP activity; induces NET formation	Level I & II: 37011073, 36228585, 32587239
Atrial Fibrillation	Gut microbiota imbalance (elevated LPS)	Epicaridial Adipose Tissue (EAT) Inflammation and Remodelling	LPS/glucose activates the NLRP3 inflammasome in EAT	Level I & II: 33757127 Level II: 35962903, 32441464

Evidence grading: Level I = Clinical trials or human cohort studies; Level II = Preclinical and mechanistic models (in vivo/in vitro); Level III = Review articles or perspectives. PMIDs classified as ‘Level I & II’ utilize integrated approaches validating preclinical mechanisms with human clinical data.

**Table 3:** Summary of Natural Phytochemicals’ Regulatory Effects on the Gut–Adipose Axis.

Phytochemical	Source	Microbial Target	Host Metabolic Benefit	Evidence Level & PMIDs
Berberine	Coptis chinensis	Inhibit TMA-producing bacteria; inhibit CutC enzyme activity	Reduce TMAO and PAGln levels; Anti-thrombotic and anti-atherosclerotic	Level II: 33863898, 38518650
Resveratrol	Grape skins, berries	It is metabolised into 4-hydroxyphenylacetic acid (4-HPA).	Activate the SIRT1 signalling pathway. Promote the browning of white adipose tissue and thermogenesis.	Level II: 39725607
Nuciferine	Lotus leaf	Reduction in the abundance of Desulfovibrio species	Repair the intestinal barrier; prevent LPS from entering the bloodstream (metabolic endotoxemia)	Level II: 33262480
Puerarin	Kudzu root	Inhibit the growth of Prevotella copri	Reduce TMA generation; alleviate atherosclerotic plaque	Level I & II:38777572
Tilianin	Dracocephalum moldavica	Regulate the composition of the gut microbiota	Upregulate the hepatic SREBP2-LDLR pathway. Improve the lipid profile.	Level II: 36610166
Hyperoside	Hypericum	Inhibition of BSH enzyme activity in microbial communities	Increase conjugated bile acids (T-β-MCA); activate the FXR-FGF15 axis	Level II: 40349961
Phytosterols	Vegetable oils, nuts	Regulate the gut microbiota composition	Reduce plasma endothelin-1 levels; improve endothelial function	Level I: 32455866

Evidence grading: Level I denotes rigorous clinical investigations (e.g., randomized controlled, double-blind trials); Level II denotes primary experimental mechanistic studies in vivo; Level I & II denotes dual validation in both preclinical models and human patients.

## Data Availability

The data that support the findings of this study are available from the corresponding authors upon reasonable, academically appropriate request. All data access requests will be reviewed and processed in compliance with institutional policies and academic research norms.
